# siRNA screening reveals that SNAP29 contributes to exosome release

**DOI:** 10.1007/s00018-023-04822-8

**Published:** 2023-06-07

**Authors:** Nina Pettersen Hessvik, Krizia Sagini, Silvana Romero, Manuel Ramirez-Garrastacho, Marta Rodriguez, Astrid Elisabeth V. Tutturen, Audun Kvalvaag, Espen Stang, Andreas Brech, Kirsten Sandvig, Alicia Llorente

**Affiliations:** 1 Department of Molecular Cell Biology, Institute for Cancer Research, Oslo University Hospital, Oslo, Norway; 2 Centre for Cancer Cell Reprogramming, Faculty of Medicine, University of Oslo, Oslo, Norway; 3 Present Address: Pathology Department, IIS-Fundación Jiménez Díaz-UAM, Center for the Biomedical Research Network in Oncology, CIBERONC, Madrid, Spain; 4 Department of Pathology, Oslo University Hospital, Oslo, Norway; 5 Department of Biosciences, University of Oslo, Oslo, Norway; 6 Department for Mechanical, Electronics and Chemical Engineering, Oslo Metropolitan University, Oslo, Norway

**Keywords:** Cholesterol, Exosomes, Small extracellular vesicles, siRNA screening, SNAP29, SNARE, Syntaxins

## Abstract

Cells release extracellular vesicles (EVs) of different sizes. Small EVs (< 200 nm) can originate from the fusion of multivesicular bodies with the plasma membrane, i.e. exosomes, and from budding of the plasma membrane, i.e. small ectosomes. To investigate the molecular machinery required for the release of small EVs, we developed a sensitive assay based on incorporation of radioactive cholesterol in EV membranes and used it in a siRNA screening. The screening showed that depletion of several SNARE proteins affected the release of small EVs. We focused on SNAP29, VAMP8, syntaxin 2, syntaxin 3 and syntaxin 18, the depletion of which reduced the release of small EVs. Importantly, this result was verified using gold standard techniques. SNAP29 depletion resulted in the largest effect and was further investigated. Immunoblotting analysis of small EVs showed that the release of several proteins considered to be associated with exosomes like syntenin, CD63 and Tsg101 was reduced, while the level of several proteins that have been shown to be released in ectosomes (annexins) or by secretory autophagy (LC3B and p62) was not affected by SNAP29 depletion. Moreover, these proteins appeared in different fractions when the EV samples were further separated by a density gradient. These results suggest that SNAP29 depletion mainly affects the secretion of exosomes. To investigate how SNAP29 affects exosome release, we used microscopy to study the distribution of MBVs using CD63 labelling and CD63-pHluorin to detect fusion events of MVBs with the plasma membrane. SNAP29 depletion caused a redistribution of CD63-labelled compartments but did not change the number of fusion events. Further experiments are therefore needed to fully understand the function of SNAP29. To conclude, we have developed a novel screening assay that has allowed us to identify several SNAREs involved in the release of small EVs.

**Supplementary Information:**

The online version contains supplementary material available at 10.1007/s00018-023-04822-8.

## Introduction

It is well established that cells release extracellular vesicles (EVs) of different origin and with different, but partially overlapping, sizes. EVs include vesicles budding from the plasma membrane (ectosomes/microvesicles) [1], apoptotic bodies released upon apoptosis [2, 3] and exosomes released upon fusion of multivesicular bodies (MVBs) with the plasma membrane [4–6]. A large heterogeneity regarding size and molecular content has been reported among different EV populations. Some of this heterogeneity is likely to be explained by different EV isolation protocols, cell type specificity and secretion mechanisms. Sequential centrifugation is one of the most common EV isolation methods, and the 100,000×*g* pellet has often been referred to as exosomes [7]. However, data support the idea that this fraction also contains small ectosomes/microvesicles, as well as material released through secretory autophagy [8–10], and therefore it might be better referred to as small EVs (sEVs).

Initially exosomes were proposed to be waste from cells [5], but they are now also considered to function in intercellular communication [6, 11, 12] and have been implicated in numerous physiological and pathological processes [13]. Several proteins and lipids have been reported to play a role in exosome release [14–16]. Interestingly, recent studies have identified endoplasmic reticulum membrane contact sites as platforms for the generation of exosomes [17, 18]. However, there are still many unanswered questions about the molecular mechanism of exosome release. Knowledge about EV release is not only of biological interest but may also have important clinical implications [19]. This is because EVs have been implicated in the development of pathophysiology, such as neurodegenerative disorders and metastasis [20]. Therefore, strategies that can interfere with the EV-mediated transfer of pathological-related molecules constitute a novel therapeutic approach.

The process leading to secretion of exosomes, which correspond to the intraluminal vesicles (ILVs) found in the lumen of MVBs, can be divided into three steps; MVB biogenesis and ILV formation, transport of MVBs to the plasma membrane and fusion of MVBs with the plasma membrane. In particular, the molecular machinery responsible for the fusion of MVBs with the plasma membrane is not completely characterized. Proteins known to be involved in membrane fusion include soluble N-ethylmaleimide-sensitive factor attachment protein receptors (SNAREs), tethering factors, Rabs, and other Ras GTPases [21–24]. SNARE proteins facilitate fusion of vesicles with their target membrane, such as the plasma membrane or the membrane of different organelles [25]. A SNARE complex is built up by three or four SNARE proteins forming four coiled-coil helices. The members of this protein family are classified as either R- or Q-SNAREs. Generally, fusion involves one R-SNARE (usually v-SNARE), and three Q-SNAREs (usually t-SNAREs) [21]. Previous studies have shown that the R-SNAREs VAMP7 (vesicle-associated membrane protein 7) and YKT6 are associated with exosome release [26–30]. Moreover, some studies have investigated the role of Q-SNAREs and showed the involvement of syntaxin (STX) 3, STX6, STX4, STX1A and SNAP23 in exosome release or in sorting of molecules to exosomes [31–37]. However, with few exceptions such as for SNAP23 and STX4 [34], these studies do not show enough evidence for a specific effect on exosome release because they analyze the 100,000×*g* pellet which, as previously mentioned, contains a mixture of sEVs formed by different mechanisms [10]. Interestingly, it has also been recently found that SNARE proteins contained in EVs function in interneuronal communication [38], and SNARE proteins in EVs are being investigated as biomarkers for neural diseases such as Parkinson’s and Alzheimer’s disease [39, 40].

To investigate in more detail the role of SNAREs in the release of sEVs, we performed an siRNA screening in the PC-3 prostate cancer cell line. To measure sEV release, we established a rapid, medium throughput and sensitive method based on the incorporation of radioactive cholesterol in vesicles. Using this novel assay, followed by standard methods for validation, we showed that the depletion of five SNARE proteins—VAMP8, SNAP29, STX2, STX3 and STX18—reduced the release of sEVs. Importantly, depletion of SNAP29 also reduced the release of sEVs from three other cancer cell lines (MCF-7, MDA-MB-231 and Caco-2), indicating that SNAP29 may play a general role in sEV secretion. Furthermore, our study suggests that it is specifically the release of sEVs containing canonical exosomal markers that is mainly affected by SNAP29 depletion.

## Materials and methods

### Materials

Ham’s F-12/ DMEM (1:1 mixture) with Glutamax, DMEM with Glutamax and RPMI were from Gibco Invitrogen (Invitrogen, Carlsbad, CA, USA). Dithiotreitol, iodoacetamide, urea and ammonium bicarbonate and OptiPrep were purchased from Sigma-Aldrich (St. Louis, MO, USA). EDTA-free protease inhibitor cocktail was from Roche Applied Science (Mannheim, Germany). Bicinchoninic acid (BCA) protein assay kit was from Pierce (Thermo Fisher Scientific, Rockford, IL, USA). 0.02 µm Anotop 25 filters were from Whatman (Dassel, Germany). CellBrite Steady 650 was from Biotium, Inc. (Landing Parkway Fremont, CA, USA). MitoTracker Red CMXRos and ProLong Gold antifade mountant with DAPI were from Invitrogen (Thermo Fisher Scientific, Rockford, IL, USA). The following primary antibodies were used: SNAP29 (Abcam, ab138500), VAMP8 (Abcam, ab89158), STX3 (Abcam, ab133750), STX2 (Millipore, ABN1010), STX18 (Proteintech, 16013-1-AP), CD9 (Abcam, ab92726), Tsg101 (BD Transduction Laboratories, 612697), Alix (Abnova, ABIN523392 and Abcam, ab117600), syntenin (Abcam, ab133267), p62 (MBL, PM045), LC3 (Cell Signaling, 2775S), annexin A1 (BD Transduction Laboratories, 610066), annexin A2 (BD Transduction Laboratories, 610068), annexin A5 (Santa Cruz Biotechnology, sc-32321), annexin A6 (Abcam, ab201024), CD63 (DSHB, H5C6), Sec61A (Abcam, ab1327), actin (Nordic BioSite, CLT9001), tubulin (Sigma-Aldrich, T5326), GAPDH (Abcam, ab9484), GM130 (BD Transduction Laboratories, 610822), Rab5 (a gift from Prof. Dr. C. Bucci, University of Salento, Lecce, Italy) and EEA1 (BD Transduction Laboratories, 610456). HRP-conjugated and Alexa Fluor-conjugated secondary antibodies were from Jackson Immunoresearch (West Grove, PA, USA). Cherry-pick library, ON-target plus siRNA, four individual siRNAs per gene, were from Dharmacon RNAi solutions (Horizon Discovery, Cambridge, UK). pCMV-Sport6-CD63-pHluorin was a gift from DM Pegtel (Addgene plasmid # 130901; http://n2t.net/addgene:130901; RRID:Addgene_130901). sn-1-O-hexadecylglycerol (HG) was from Santa Cruz Biotechnology (Dallas, TX, USA) and manumycin A from Sigma-Aldrich (St. Louis, MO, USA). [^14^C]cholesterol was from PerkinElmer (Shelton, CT, USA). Lysylendopeptidase (Lys-C) was from Wako (Neuss, Germany) and trypsin (mass spectrometry grade) was from Promega (Madison, WI, USA). Acetonitrile and formic acid were both obtained from Fluka Analytical (Sigma-Aldrich, St. Louis, MO, USA). For mass spectrometric analysis Empore Extraction Disk (Varian, St. Paul, MN) was used for desalting of the samples prior to analysis on a Q Exactive hybrid quadropole-orbitrap plus interfaced with an EASY Spray PepMapRSLC column (C18, 50 cm bed length, 2 μm, 100 Å, 75 μm inner diameter (Thermo Fisher Scientific, Waltham, MA) for peptide separation.

### Cell culture

The human prostate cancer epithelial cell line PC-3 was obtained from ATCC (Manassas, VA, USA). PC-3 cells were cultured in Ham’s F-12/ DMEM (1:1 mixture) with Glutamax supplemented with 7% foetal calf serum (FCS), 100 units/ml penicillin and 100 units/ml streptomycin, in a humidified 5% CO_2_ atmosphere at 37 °C. The breast cancer cell lines MCF-7 and MDA-MB-231, and the colorectal adenocarcinoma cells Caco-2 were also from ATCC. MCF-7 and MDA-MB-231 cells were cultured in RPMI-1640 with l-glutamine, supplemented with 10% FCS. The Caco-2 cells were cultured in DMEM with Glutamax, supplemented with 15% FCS. The MCF-7, MDA-MB-231 and Caco-2 were also supplemented with 100 units/ml penicillin and 100 units/ml streptomycin and grown in a humidified 5% CO_2_ atmosphere at 37 °C.

### siRNA transfection

ON-target plus individual siRNAs against the 49 genes listed in Supplementary Table S1 (25 nM) and non-targeting siRNA (control, 25 nM) were delivered to the cells using Lipofectamine RNAiMAX transfection reagent (Life Technologies), according to the manufacturer’s protocol. Four individual siRNAs were used per gene (Supplementary Table S2). For the verification experiments ON-target plus individual siRNAs against five genes—VAMP8, SNAP29, STX2, STX3 and STX18—were used with three individual siRNAs per gene using the same transfection protocol. Cells were lysed at the end of the experiments to measure the knockdown efficiency.

### Screening assay for sEV release

**Fig. 1:**
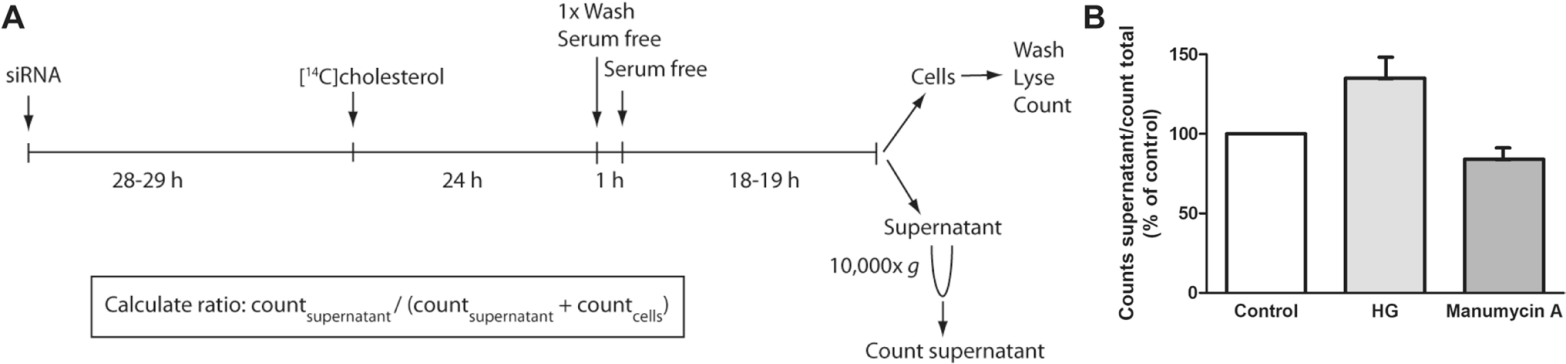
Experimental setup for [^14^C]cholesterol-based screening assay for sEV release. **A** PC-3 cells were transfected with ON-target plus individual siRNAs (25 nM). The next day, cells were radiolabeled with [^14^C]cholesterol (0.1 µCi/ml, 2 µM) for 24 h, washed, and incubated with serum-free medium for 19 h. The medium was collected and centrifuged for 30 min at 10,000×*g*, before the supernatant was counted using a β-counter. Cells were washed, lysed and counted using a β-counter. The level of cholesterol in sEVs was estimated as the percentage of radioactivity in the medium relative to the total radioactivity in the medium and cells. **B** Experiments showing the effect of HG (20 µM) and manumycin A (250 nm) on the level of EV-associated [^14^C]cholesterol. The experiments were performed 4 times with duplicates

Cells were seeded in 12-well plates and transfected with ON-target plus individual siRNAs as described above. The next day, cells were radiolabeled with [^14^C]cholesterol (0.1 µCi/ml, 2 µM) in complete Ham’s F-12/ DMEM (with 7% FCS) for 24 h (Fig. 1). Subsequently, cells were washed with serum-free medium, and incubated for 1 h with serum-free medium. Thereafter, the cells were incubated with serum-free medium for 18–19 h. The medium was collected and centrifuged for 30 min at 10,000×*g* to remove cells and large vesicles, and the supernatant was counted in a β-counter. Cells were washed with PBS, lysed in 0.1 M KOH and counted using a β-counter. sEV-associated cholesterol was estimated as percentage of radioactivity in the supernatant relative to the total radioactivity (supernatant and cells). In some experiments the cells were treated with HG (20 µM) or with manumycin A (250 nM) for two days before radiolabeled cholesterol was quantified.

### Cell treatment and isolation of small EVs by sequential centrifugation

Two days after transfection, PC-3 cells grown on 10 cm plates were washed twice with serum-free medium and incubated for 18–19 h in serum-free medium to collect sEVs. For MDA-MB-231 and Caco-2 cells, the vesicles were collected for 24 h starting 2 days after transfection. For MCF-7, the vesicles were collected for 42–44 h starting 1 day after transfection. sEVs were isolated from the conditioned media of cells as previously described [41]. We have previously characterized sEVs isolated by this method using electron microscopy, nanoparticle tracking analysis and immunoblotting analysis [42–44]. Briefly, the medium was centrifuged at 1000×*g* for 10 min, thereafter the supernatant was centrifuged at 10,000×*g* in an MLA80 rotor for 30 min to remove cell debris and large ectosomes/microvesicles. The supernatant was then centrifuged at 100,000×*g* in a MLA80 rotor for 70 min. The pellet was washed with PBS, and centrifuged again at 100,000×*g* for 70 min. The pellets were then resuspended in equal volumes of PBS or lysis buffer before further analyses. All centrifugation steps were carried out at 4 °C.

### OptiPrep density gradient separation

sEVs (100,000×*g* pellet) were resuspended in PBS and mixed with iodixanol/PBS for a final 30% iodixanol solution. The suspension was added to the bottom of a tube and solutions of descending concentration of iodixanol in PBS (30%, 24%, 18%, 12% and 6%) were carefully layered on top yielding the complete gradient. Identical gradients without sample were generated in the same manner for later determination of fraction densities. The bottom-loaded 6–30% gradients were subjected to ultracentrifugation at 100,000×*g* for 20 h at 4 °C using a SW40 Swinging Bucket rotor. After centrifugation, starting from the top of the tube, 12 individual gradient fractions (1 ml each) of increasing density were collected and diluted with 5 ml PBS, followed by centrifugation at 100,000×*g* for 70 min at 4 °C. The pellets were resuspended in equal volumes of lysis buffer and used for further analyses.

### Preparation of cell lysates

PC-3 cells were washed with cold PBS and total cell lysates were prepared in lysis buffer (50 mM Tris–HCl, 300 mM NaCl, 1 mM EDTA, 0.5% Triton X-100, pH 7.4) in the presence of an EDTA-free protease inhibitor mixture. The suspension was incubated on ice for 20 min and centrifuged at 20,000×*g* for 10 min at 4 °C. The supernatant was collected and stored at −20 °C.

### Nanoparticle tracking analysis (NTA)

The concentration and the size distribution of the sEV samples were measured by NTA. sEV pellets were resuspended in the amount of PBS (filtered through a 0.02 µm Anotop 25 filter) needed to obtain a concentration within the recommended range for NTA analysis in a Nanosight NS500 instrument (2 × 10^8^–1 × 10^9^ particles per ml), and vortexed for 1 min. The samples were then loaded into a NS500 instrument (Malvern Instruments Ltd, Worcestershire, UK). Five videos, each of 60 s, were acquired for every sample. Videos were subsequently analyzed with the NTA 3.1 software, which identifies and tracks the center of each particle under Brownian motion to measure the average distance the particles move on a frame-by-frame basis.

### Protein measurements

The amount of protein was determined using a BCA assay kit according to the manufacturer’s instructions.

### SDS-PAGE and immunoblotting

sEVs were lysed in lysis buffer (50 mM Tris–HCl, 300 mM NaCl, 1 mM EDTA, 0.5% Triton X-100, 0.2% Lauryl (SDS), pH 7.4). Cells were lysed as described above. sEVs and cell lysates were mixed with loading buffer, separated by 4–20% SDS-PAGE and transferred to PVDF membranes. The membranes were blocked in 5% milk for 60 min, followed by overnight incubation with the indicated primary antibodies in 5% BSA. Then, they were incubated for 60 min with HRP-conjugated secondary antibodies, and the signals were finally detected with SuperSignal West Dura Extended Duration Substrate (Thermo Scientific) in a ChemiDoc Imaging System (Bio-Rad).

### Electron microscopy

For negative staining, sEVs were placed onto formvar/carbon coated grids for 5 min, washed twice with PBS and four times with ultrapure water, and then stained with 4% uranyl acetate for 3 min. After a wash in water, grids were dried for 10 min before observing samples on a JEOL-JEM 1230 at 80 kV. Images were recorded with a Morada digital camera and further image processing performed with ImageJ and TEM ExosomeAnalyzer [45]. The minimal object diameter for particle counting using TEM ExosomeAnalyzer was set at 30 nm. Cells for cryo-sectioning and immuno-EM were fixed with 4% methanol-free paraformaldehyde and 0.1% glutaraldehyde (Electron Microscopy Sciences, Hatfield, PA) in 0.2 M HEPES, and prepared for cryo immuno-electron microscopy basically as described in [46]. Labeling for CD63 was done using mouse anti-CD63 (H5C6, DSHB) followed by rabbit anti-mouse IgG (Cappel Research Reagents, ICN Biochemicals, Irvin, CA) and colloidal gold coated with protein A (G. Posthuma, Utrecht, The Netherlands). Sections were examined using a Tecnai G2 Spirit TEM (FEI, Eindhoven, The Netherlands). Images were processed using Adobe Photoshop.

### Immunofluorescence microscopy

Cells grown onto glass coverslips were fixed with 4% paraformaldehyde and permeabilized with 0.05% saponin before incubation with the primary antibodies. After incubation with the secondary antibodies conjugated with Alexa 488, Alexa 555 or Alexa 568, cells were washed and mounted with ProLong Gold antifade mounting medium containing DAPI. The cells were imaged either using a Zeiss LSM710 laser scanning confocal microscope (Carl Zeiss MicroImaging GmbH, Jena, Germany) equipped with an Ar-Laser multiline (458/488/514 nm), a DPSS-561 10 (561 nm), a Laser diode 405–30 CW (405 nm), and a HeNe-laser (633 nm) or a Nikon ECLIPSE Ti2-E microscope (Nikon Corp, Tokyo, Japan) equipped with a CSU-W1 spinning disk confocal unit (50 µm pinhole size, Yokogawa Electric Corp, Tokyo, Japan), a Prime BSI sCMOS camera (Teledyne Photometrics, Tucson, AZ, US), 405 nm, 488 nm, 561 nm and 638 nm lasers, BrightLine bandpass filters (447/60, 525/50, 600/52 and 708/75). The objective used was a Zeiss Plan-Apochromat 63x/1.40 Oil DIC M27 for LSM710 or a CFI Plan Apo λ 100x (NA 1.54 Oil) for ECLIPSE Ti2-E. Images were acquired using the ZEN 2010 software (Carl Zeiss MicroImaging) for LSM710 or NIS-Elements AR 5.30 software (Nikon Instruments Inc.) for ECLIPSE Ti2-E. Multichannel images of at least 7 random fields of view in each coverslip were captured. For each field of view, 5–15 optical Z-sections were acquired with Z-section spacing 0.4 µm. Image processing and analysis was done with LSM710 software, Fiji (National Institutes of Health), Adobe Illustrator (Adobe) and IMARIS (Oxford Instruments Technology). Imaris was used to analyze colocalization.

### Analysis of MVB fusion events at the plasma membrane using CD63-pHluorin

The analysis of MVB fusion events at the plasma membrane was performed as in Verweij et al. with few modifications [34]. PC-3 cells grown in MatTek 3.5 cm dishes were transfected with SNAP29-5 and non-targeting siRNA as described in the siRNA transfection section. The medium was replaced 12 h after transfection to remove the transfection reagent and then cells were transfected with 1 μg CD63-pHluorin plasmid using Fugene 6 with 3:1 ratio of reagent to DNA. Live-cell imaging was performed 48 h after CD63-pHluorin transfection on a Nikon ECLIPSE Ti2-E microscope (Nikon Corp, Tokyo, Japan) using a CFI Plan Apo λ 100× (NA 1.54 Oil) objective, a CSU-W1 spinning disk confocal unit (50 µm pinhole size, Yokogawa Electric Corp, Tokyo, Japan), a Prime BSI sCMOS camera (Teledyne Photometrics, Tucson, AZ, US), 405 nm, 488 nm, 561 nm and 638 nm lasers, BrightLine bandpass filters (447/60, 525/50, 600/52, and 708/75), and NIS-Elements AR 5.30 software. Environmental control was provided by a stage-top incubator with temperature control, digital CO_2_ control, and active humidification (Okolab). Images of at least 5 random fields of view per condition were captured. Images were acquired at 1 Hz focusing at or near the plasma membrane. Image analysis was performed using the ImageJ2/Fiji plugin ExoJ [47]. On average, ≥ 10 cells were imaged per condition in ≥ 6 different fields of view.

### In-solution digest

For mass spectrometry analysis, sEVs were isolated from the conditioned serum-free media using two 15 cm plates per condition. Proteins were then precipitated by incubating the samples with ice cold acetone with 1 M HCl (four times the sample volume; 80 µl to 20 µl sample) overnight at −20 °C. Samples were centrifuged at 13,000×*g* at 4 °C for 15 min, and the pellets were vacuum dried to remove traces of acetone before being dissolved in 50 µl 6 M urea in 100 mM ammonium bicarbonate and reduced with 10 mM dithiotreitol at 30 °C for 30 min. Exposed side chains were alkylated by incubation with 25 mM iodoacetamide for1 h at room temperature protected from light. The enzymatic digestion was initiated by adding 0.5 µg Lys-C to the samples and incubating them at 37 °C for 2 h. Finally, 50 mM ammonium bicarbonate (240 µl) with 0.5 µg of trypsin was added and the samples were incubated first for 1 h at 37 °C, followed by overnight incubation at 30 °C. Prior to nLC–MS/MS analysis, the obtained peptides were desalted by reversed-phase chromatography using C18 micro columns prepared by stacking three layers of C18 Empore Extraction Disk into 200-μL pipette tips. Peptides were eluted by applying 80% acetonitrile in 0.1% formic acid. Acetonitrile was evaporated in a vacuum drier, and the volume was adjusted by adding 0.1% formic acid.

### Mass spectrometric analyses

Samples were analyzed on a Q Exactive hybrid quadropole-orbitrap plus interfaced with an EASY-nLC 1000 (Thermo Fisher Scientific). Peptides were separated on a 50-cm bed length EASY Spray PepMapRSLC column (C18, 2 μm, 100 Å, 75 μm inner diameter) with column temperature 60 °C and a flow rate of 300 nl/min. Solvent A was water with 0.1% formic acid and solvent B was acetonitrile with 0.1% formic acid. A 60 min gradient was used for separation: 2% to 7% B in 5 min and 7% to 30% in 50 min. The mass spectrometer was operated in a data-dependent acquisition mode with automatic switching between MS and MS/MS. Full MS scans were acquired in the resolution of 70.000 at m/z 200 with automatic gain control target value of 3 × 10^6^ or maximum injection time of 100 ms, within the scan range of 400–1200 m/z. Peptide fragmentation was performed by higher energy collision dissociation with normalized collision energy set to 25. MS/MS spectra were acquired of the ten most abundant ions (Top10 method) with a dynamic exclusion time of 30 s. MS/MS scans were acquired with a resolution of 17.500 with automatic gain control target value of 1 × 10^5^ or maximum injection time of 100 ms. Ion selection was performed within an isolation window of 3.0 m/z and fixed first mass was set to 100 m/z. Underfill ratio was 1.0% and intensity threshold 1.0e^4^.

### Database search

The software package MaxQuant version 1.6.1.0 [48] with the inbuilt Andromeda search engine [49] was used for protein identification and label-free quantitation. MS and MS/MS spectra were searched against the UniProtKB FASTA database for the human proteome (85,915 entries including isoforms and canonical sequences; downloaded from http://www.UniProt.org, October 2017). The following parameters were applied: enzyme: trypsin with no proline restriction; variable modifications: oxidation, acetylation (protein N-term), fixed modification: carbamidomethyl (C). The first search was performed with mass tolerance of 20 ppm for the precursor ion, and after recalibration, a 4.5-ppm tolerance was used in the main search; mass tolerance for fragment ions was set to 20 ppm. Minimal unique peptides were set to 1, and a false-discovery rate of 0.01 was used in all instances. For identification, match between runs was enabled, and the MaxQuant label-free quantification algorithm with a minimum ratio count of one was used for quantification. The mass spectrometry proteomics data have been deposited to the ProteomeXchange Consortium via the PRIDE [50] partner repository with the dataset identifier PXD040958.

### Data processing

For proteomic analysis, statistical analysis was carried out using the Perseus software package version 1.6.0.7 [51]. Proteins considered by MaxQuant to be possible contaminants, hits from reverse sequences or those only identified by site were removed from the identification lists prior to analysis. The remaining data was log2 transformed. sEV samples were grouped into two groups, SNAP29 siRNA and non-targeting siRNA, from which only protein hits with LFQ intensity values > 0 in more than 70% of the samples in the two groups were included in the downstream analysis. An imputation approach was used to replace the zero LFQ values by random numbers drawn from the normal distribution of the data to simulate the distribution of low abundant proteins. Paired Student’s t-test was performed using a truncation based on permutation-based FDR < 0.05 for correction of multiple testing, allowing 250 randomizations for both groups.

Quantification of immunoblotting was performed using Fiji software. All experiments were carried out using duplicates and run in at least three independent experiments. Statistical analysis was carried out by using a paired two-tailed t-test. A P-value < 0.05 was considered significant.

For imaging data analysis, data were analyzed using IMARIS to determine Pearson’s correlation coefficients and signal distribution in cells. On average, ≥ 30 cells were imaged in ≥ 10 different fields of view. A P-value < 0.05 was considered significant. CD63 puncta were detected as fluorescence area having a minimum surface of 0.2 μm^2^. Z-sections were projected by the sum slices projection method and background subtraction was performed using Fiji. On average, ≥ 15 cells were imaged in ≥ 10 different fields of view. A P-value < 0.05 was considered significant.

## Results

### Establishment of a radioactive assay for screening of sEV release

EVs are often isolated from conditioned media by sequential centrifugation, which is a relatively time-consuming and low throughput method. In addition, several of the tools used for EV analysis require large numbers of cells to get detectable signals. This approach is not optimal for screening studies and we therefore decided to establish a novel [^14^C]cholesterol-based screening assay (Fig. 1a). The assay is based on the high amounts of cholesterol in sEV membranes [52], and the high sensitivity of radioactive labeling to detect molecules. In this assay, most of the radioactive signal is expected to be associated with sEVs after removal of cells, cell debris and large vesicles from the conditioned medium by centrifugation at 1000×*g* first and then at 10,000×*g*. Therefore, the radioactivity associated to this solution provides an estimation of the amount of sEVs released by cells. EV-associated cholesterol was then estimated as the percentage of radioactivity in the medium (after 10,000×*g* centrifugation) compared to the total radioactivity in the cells and the medium (after 10,000×*g* centrifugation). Using this calculation, we found that approximately 2–4% of the total [^14^C]cholesterol signal was found in the conditioned media of PC-3 cells. To support the validity of the method, we selected compounds that previously have been shown to affect sEV release with conventional methods in the specific cell line used in our study. PC-3 cells were therefore treated with the ether lipid precursor HG or manumycin A, which have respectively been shown to increase and decrease sEV release in these cells, [44, 53]. As shown in Fig. 1b, the amount of [^14^C]cholesterol in the medium was higher in HG-treated cells and lower in manumycin A-treated cells compared to untreated cells. These control experiments indicate that the assay works as intended.

### Identification of proteins involved in sEV release

**Fig. 2:**
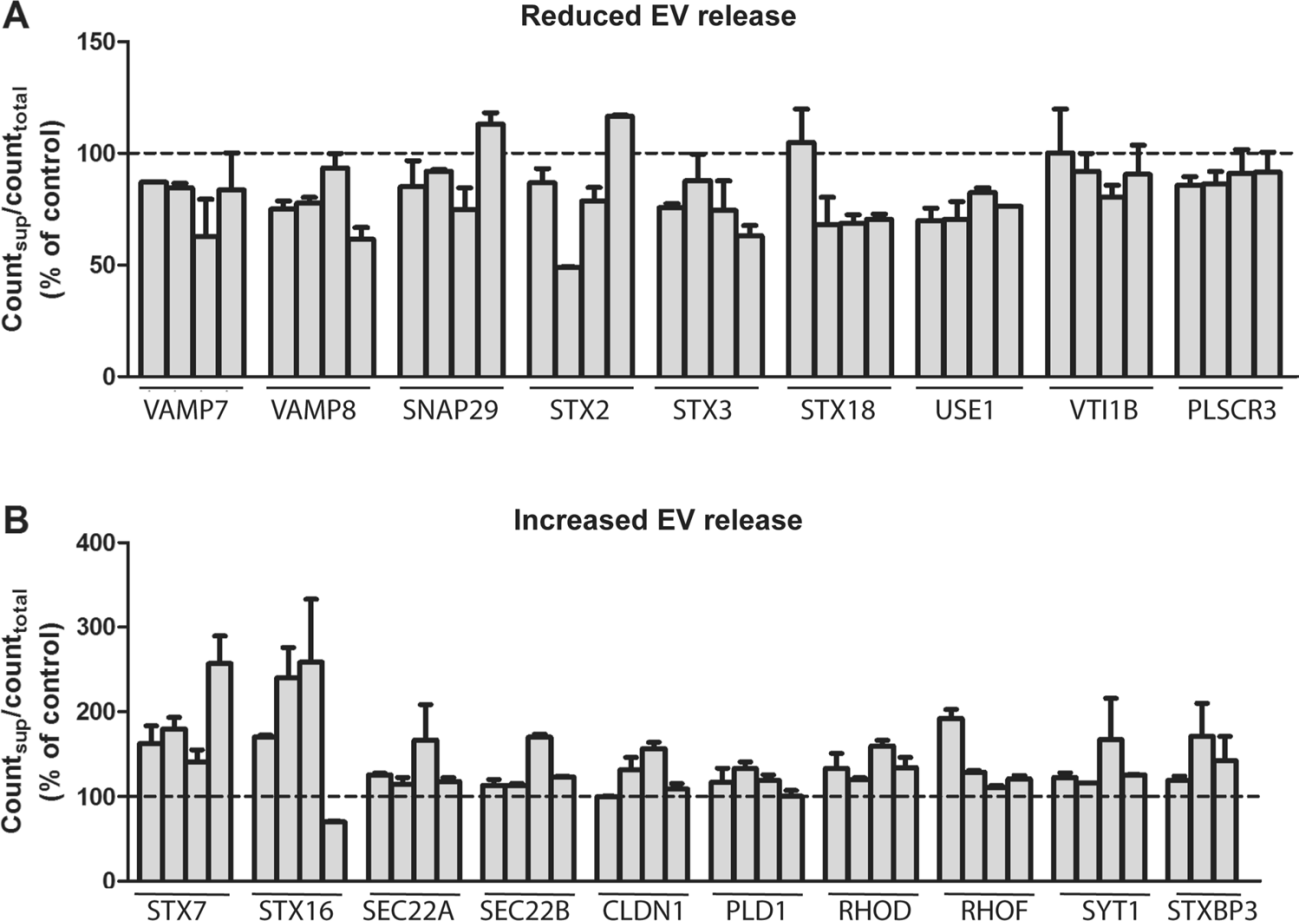
Proteins identified by the screening assay as candidates that (**A**) reduce or (**B**) increase sEV secretion after their knockdown. The error of the controls was 8% (n = 6; samples in each experiment in quadruplicate)

Despite the relevance of EVs in pathophysiological conditions, the molecular mechanisms responsible for their secretion are not completely understood. In order to get additional information about this process, we performed an siRNA screening and measured sEV secretion using our [^14^C]cholesterol-based assay. For the screening, PC-3 cell growing in 12-well plates were transfected with siRNA against 49 genes (Table S1), using four different siRNAs against each gene (Table S2). The majority of the selected genes code for SNARE proteins, but several small GTPases, the phospholipases C and D, the tight junction proteins claudins, phospholipid scramblases, sorting nexins, and several PDZ domain containing proteins, including syntenin-1, were also included. Some of these proteins have been shown to affect sEV release in other cell lines (Rab27, syntenin, VAMP7, PLD2, YKT6, SNAP23 and STX4) [26, 27, 30, 33, 54–57]. The criteria that we used to select proteins affecting sEV release was that at least three of the four siRNAs against the same protein showed an effect in the same direction. The results of the screening showed that the depletion of nine proteins (VAMP7, VAMP8, SNAP29, STX2, STX3, STX18, USE1, VTI1B and PLSCR3) reduced sEV secretion (Fig. 2a). In addition, depletion of ten proteins (STX7, STX16, SEC22A, SEC22B, CLDN1, PLD1, RHOD, RHOF, SYT1 and STXBP3) was shown to increase sEV secretion (Fig. 2b). It should be mentioned that we did not establish a threshold effect in these experiments, and that the effects of several of these proteins in sEV release were relatively small. Moreover, as discussed later, it is important to be aware of the limitations of this assay and the need to use alternative methods to reach final conclusions.

**Fig. 3:**
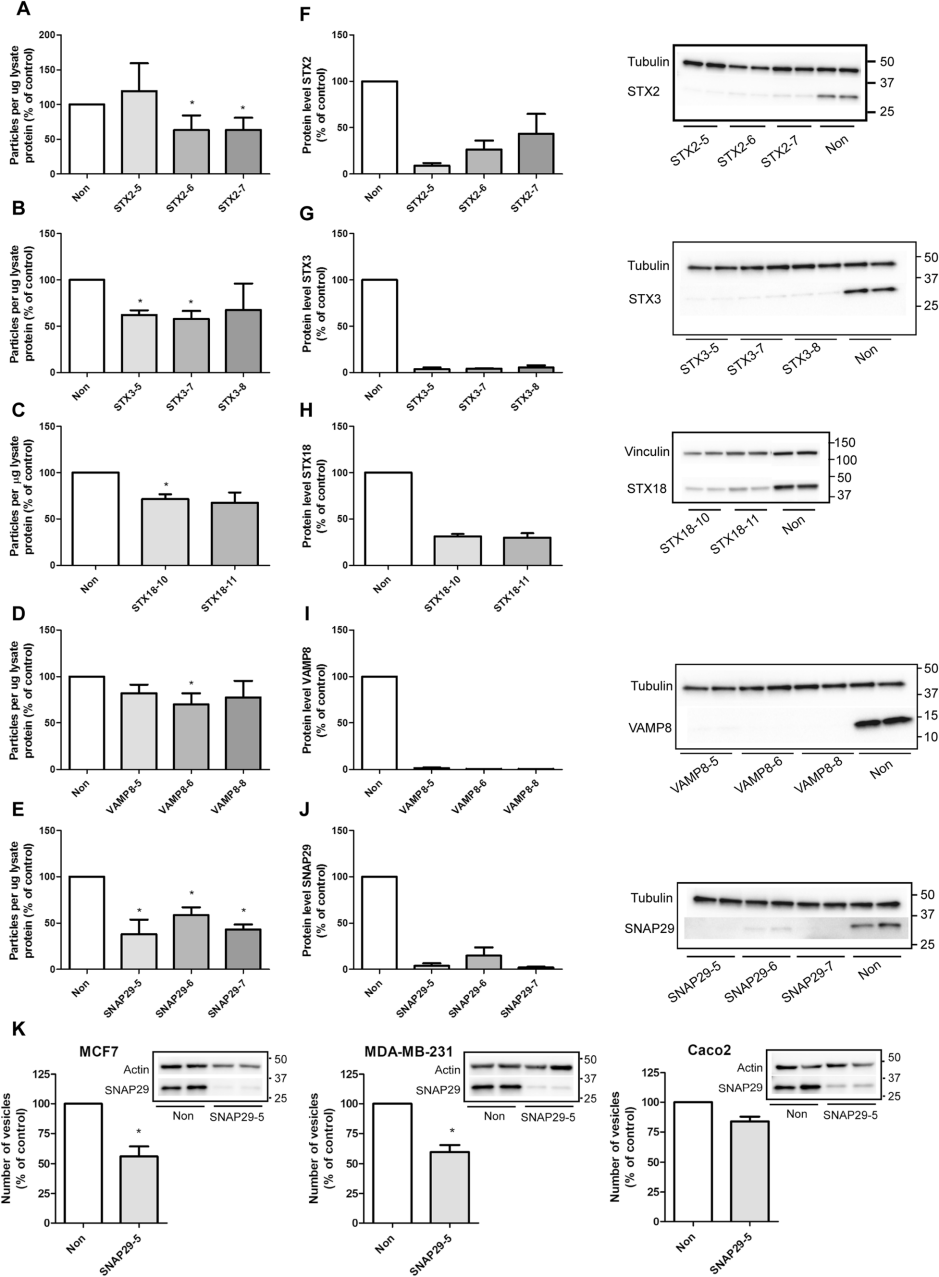
Depletion of STX2, STX3, STX18, VAMP8 and SNAP29 reduce the release of sEVs. sEVs were isolated by sequential centrifugation and their concentration measured by NTA after depletion of (**A**) STX2, (**B**) STX3, (**C**) STX18, (**D**) VAMP8 and (**E**) SNAP29. Knockdown efficiency was measured by immunoblotting 3 days after transfection with siRNA (25 nM) against (**F**) STX2, (**G**) STX3, (**H**) STX18, (**I**) VAMP8 and (**J**) SNAP29. **A–J** Data shows mean ± SEM from 3–4 independent experiments. *P < 0.05 versus non-targeting control (non). **K** sEVs were isolated from MCF-7, MDA-MB-231 and Caco-2 cells by sequential centrifugation after depletion of SNAP29 by siRNA (25 nM). For MDA-MB-231 and Caco-2 cells, vesicles were collected for 24 h, starting 2 days after transfection. For MCF-7, vesicles were collected for 42–44 h, starting 1 day after transfection. Particles in the 100,000×*g* pellet were measured by NTA. Knockdown efficiency 3 days after transfection was measured by immunoblotting, using actin as control. Experiments were performed twice (Caco-2) or 3 times (MCF-7, MDA-MB-231) in duplicate

Given the role of SNAREs in facilitating membrane fusion and their possible implication in the fusion of MVBs with the plasma membrane, we chose to validate the effect of the SNAREs that reduced sEV secretion, i.e. VAMP8, SNAP29, STX2, STX3 and STX18 (Fig. 2a). VAMP7 depletion also caused a reduction of sEV release, but this protein has previously been reported to reduce sEV secretion in other cell lines and was not further investigated here [26, 30]. For validation, sEVs released from control and knockdown (kd) cells were isolated by sequential centrifugation and pelleted at 100,000×*g* [41]. The pellet was then resuspended in PBS, and the number of particles was measured by nanoparticle tracking analysis (NTA). In terms of syntaxins, depletion of STX2 by two siRNAs significantly reduced the number of released vesicles by 37%, whereas the third did not have any effect (Fig. 3a). Depletion of STX3 also reduced the secretion of vesicles by 32–42% (Fig. 3b). In particular, two of the siRNAs used gave a statistically significant reduction in the number of released particles, whereas the third one showed the same tendency. In addition, depletion of STX18 by two different siRNAs reduced the release of sEVs by 28–33%, though only one of them was statistically significant (Fig. 3c). The effect of VAMP8 and SNAP29 on sEV release was investigated next. As shown in Fig. 3d, the release of sEVs was reduced by 18–30% after VAMP8 depletion with three siRNAs, though only one siRNA gave statistically significant reduction. Finally, depletion of SNAP29 gave the strongest reduction in the number of released sEVs, and all the three tested siRNAs resulted in a statistically significant reduction by 41–62% (Fig. 3e).

Control experiments showed that knockdown was highly efficient for all the siRNA: 57–91% for STX2 (Fig. 3f), 94–96% for STX3 (Fig. 3g), close to 70% for STX18 (Fig. 3h), 98–99% for VAMP8 (Fig. 3i) and 85–98% for SNAP29 (Fig. 3j). Moreover, the diameter of approximately 80% of the detected particles was in the 100–175 nm range (Fig.S1), and the particle size distribution was not significantly changed after depletion of these SNAREs (Fig.S1).

In conclusion, the results from the [14C]cholesterol-based screening study were verified for the selected proteins using gold standard techniques for sEV isolation and analysis. Based on these results we chose to investigate the role of SNAP29 in more detail because its depletion gave the strongest reduction in the number of released particles in PC-3 cells (Fig. 2e). Furthermore, we found that SNAP29 depletion also reduced sEV release in the breast cancer cell lines MCF-7 and MDA-MB-231 and in the colorectal adenocarcinoma Caco-2 cells, when measured by NTA (Fig. 3k). This result suggests that SNAP29 may play a general role in sEVs secretion.

### Analysis of SNAP29 depletion in sEVs release by electron microscopy

**Fig. 4:**
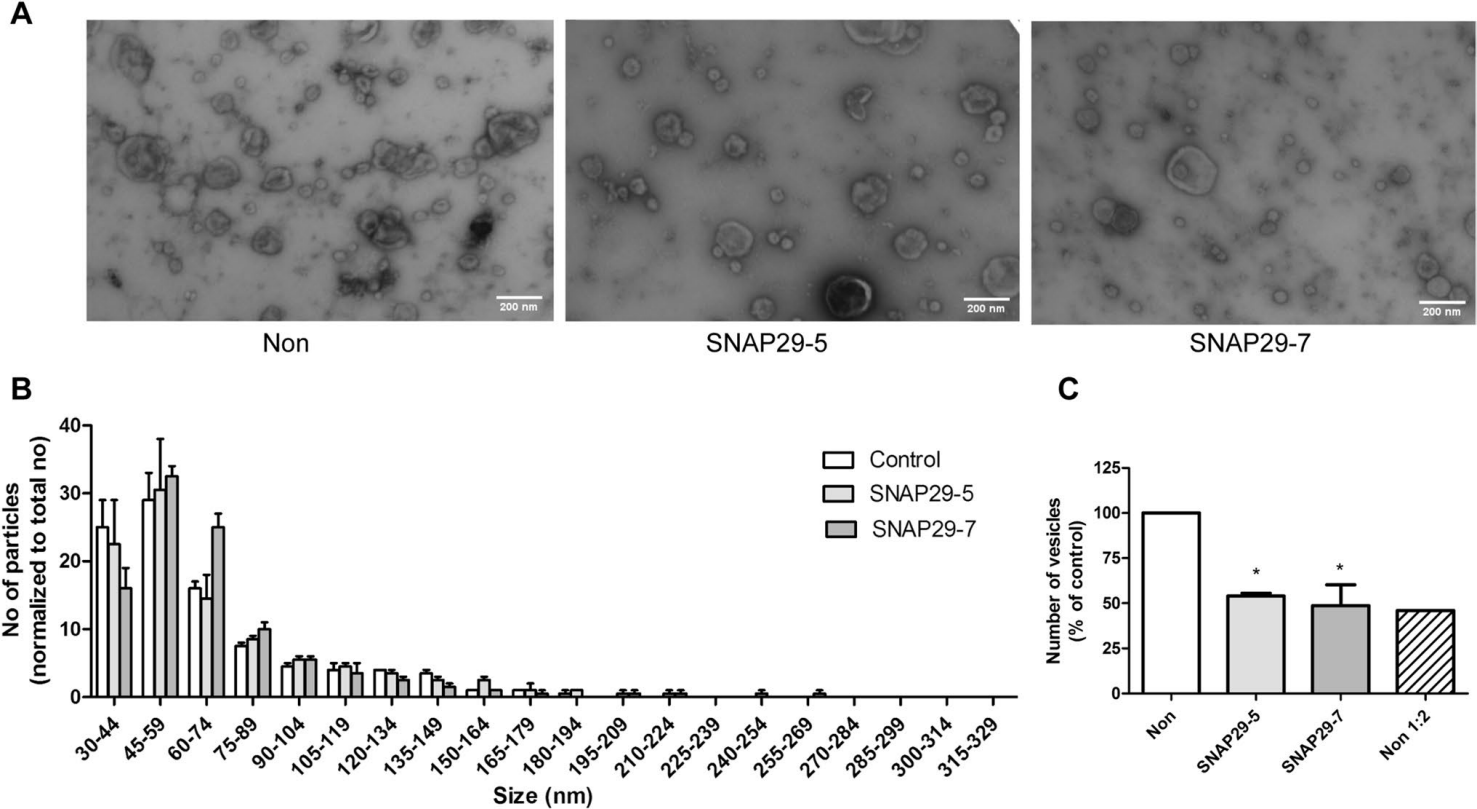
EM analysis of sEVs from SNAP29 depleted and control PC-3 cells. sEVs were isolated by sequential centrifugation, placed onto formvar/carbon coated grids, washed, stained with 4% uranyl acetate and imaged on a JEOL-JEM 1230 at 80 kV. **A** Representative images of sEVs from SNAP29 depleted and control (non) cells. Size bar (200 nm) is indicated. **B** Size distribution of sEVs shown as number of particles normalized by total number of particles for each condition. Five images per condition were analyzed by TEM ExosomeAnalyzer. The data shows mean + SEM from three independent experiments. **C** Number of particles shown as percentage of control. Total number particles were counted in the same images used to prepare **B**. The data shows mean + SEM from three independent experiments

Electron microscopy (EM) allows the visualization of EV morphology and is also useful to detect possible contaminants in EV samples. Electron micrographs in Fig. 4a showed that both control and SNAP29 kd PC-3 cells release vesicles of different size and morphology. In addition, the size distribution of the vesicles was similar in control and SNAP29 kd cells (Fig. 4b), having approximately 70% of the counted vesicles a diameter in the 30–75 nm range. Interestingly, in agreement with the NTA data, quantification of the number of vesicles on the electron micrographs showed a reduced number of vesicles after SNAP29 depletion (Fig. 4c). EM microscopy has not been much used for vesicle quantification, but our control experiments show that the amount of EVs quantified by EM is proportional to the sample dilution (Fig. 4c).

### SNAP29 depletion reduces the secretion of sEVs carrying classical exosomal markers

**Fig. 5:**
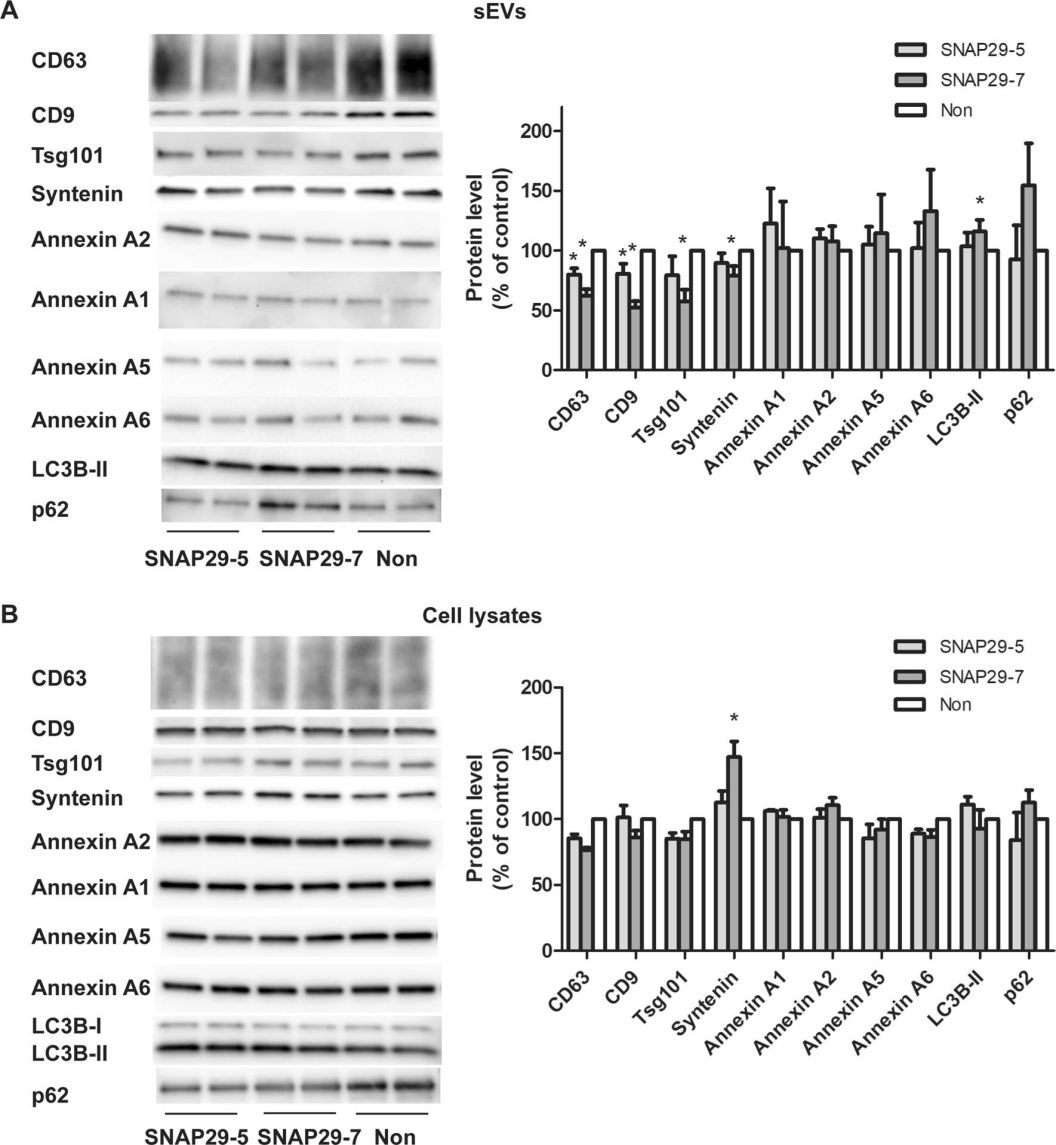
Depletion of SNAP29 decreases the secretion of exosomal proteins, but not of annexins and autophagy-related proteins. sEVs were isolated by sequential centrifugation from SNAP29-depleted PC-3 cells and lysed. Releasing cells were also lysed and equal volumes of sEV or cell lysate loaded on SDS-PAGE gels. **A** Representative immunoblots and quantification of sEV proteins. **B** Representative immunoblotting and quantification of cellular proteins. Data shows mean ± SEM, n = 3–5. *P < 0.05 versus non-targeting control (non)

sEVs of different origin can be pelleted at 100,000×*g* [10]. To investigate whether SNAP29 depletion affected a specific sEV population, we first analyzed the level of proteins commonly associated with exosomes by immunoblotting. We also measured the levels of some annexins, since annexin A1, A2 and A5 have been reported to be absent from classical exosomes (containing CD63, CD81, CD9, flotillin, syntenin, Tsg101 and Alix) and present on sEVs derived from the plasma membrane [10]. As shown in Fig. 5a the level of the exosomal related markers Tsg101, syntenin, CD63, and CD9 in the 100,000×*g* pellet was reduced after SNAP29 depletion, but not the level of annexin A1, annexin A2, annexin A5 and annexin A6. The level of the same proteins was investigated also in control and SNAP29-depleted cell lysates to exclude that the variations observed in sEVs were due to altered cellular levels (Fig. 5b). In conclusion, these results suggest that depletion of SNAP29 reduces the release of exosomes rather than ectosomes.

SNAP29 has been associated to unconventional protein secretion through autophagy, also known as secretory autophagy [58]. In addition, material released by secretory autophagy may be pelleted at 100,000×*g* [59, 60]. Therefore, we also investigated whether the reduction of particles observed after SNAP29 depletion could, at least partly, be explained by reduced secretory autophagy. However, we found that the level of the autophagic markers LC3B and p62 in the 100,000×*g* fraction did not change or was slightly increased after SNAP29 depletion (Fig. 5a). This is not the case in HEK-293 T cells, where it has been shown that there is an increased release of LC3B, p62 and other autophagy related proteins in SNAP29 depleted cells compared to control cells [60, 61]. It should also be noted that it is only the lipidated form of LC3B that appears in the sEV fraction [9]. Since this LC3B form is not present at the plasma membrane, its presence in the 100,000×*g* pellet suggests that it originates from endocytic/autophagic organelles. Control analysis of LC3B and p62 in the lysates did not show significant differences after SNAP29 depletion (Fig. 5b).

**Fig. 6:**
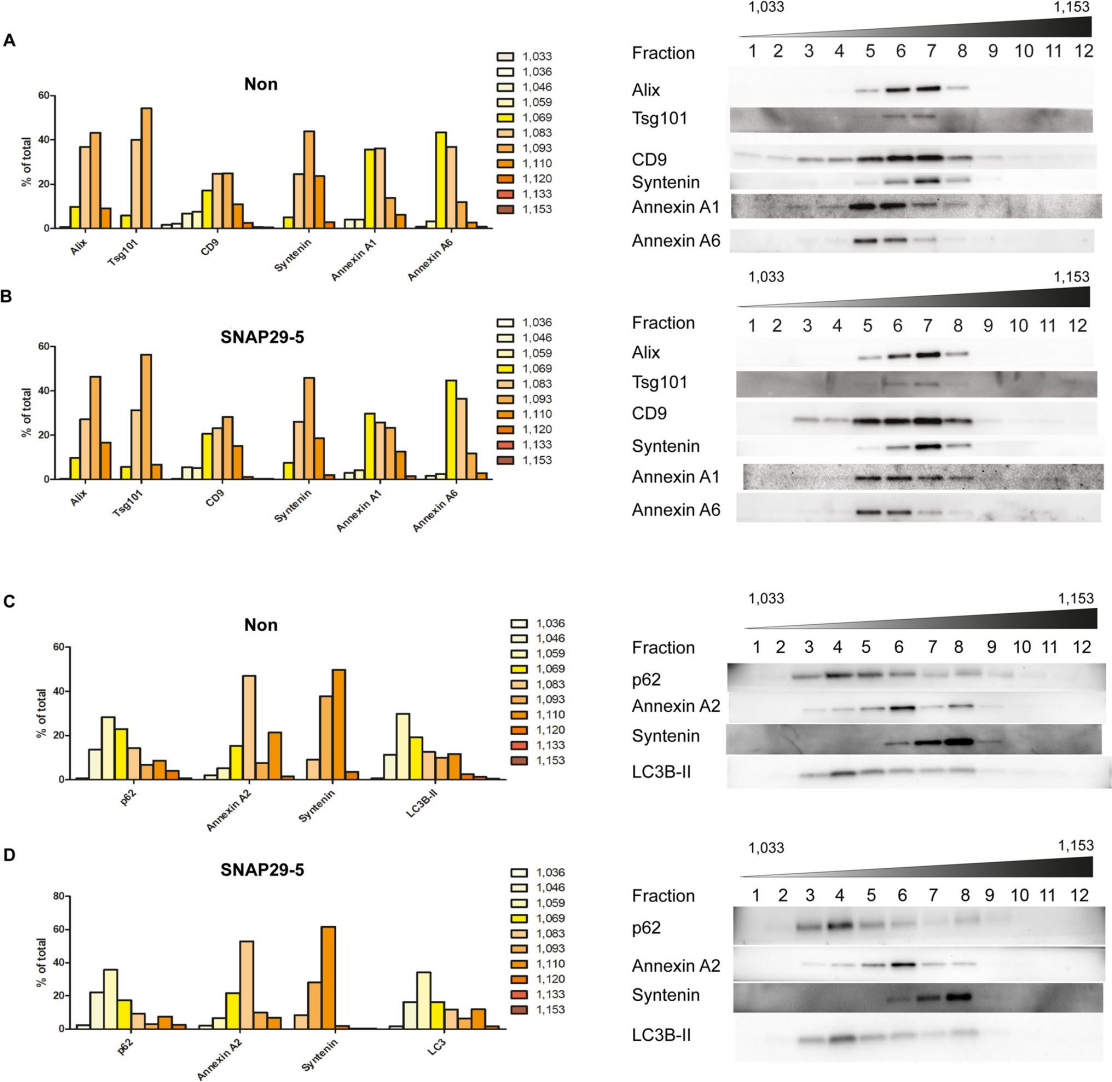
The sEV fraction contains material of different density and protein composition. sEVs from (**A, C**) control and (**B, D**) SNAP29-depleted PC-3 cells were separated in a bottom-loaded OptiPrep density gradient (6–30%) by centrifugation at 100,000×*g* for 20 h. Twelve fractions were collected and immunoblotting was used to detect exosomal proteins, annexins and autophagy-related proteins. Equal volumes were loaded on SDS-PAGE gels. Experiments were performed 3 times, representative immunoblottings and quantification are shown. Band intensity is shown as % of signal per fraction normalized by the total signal for each protein

The results presented above suggest that depletion of SNAP29 reduces the release of exosomes. In order to investigate whether classical exosomal markers, annexins and autophagy markers were present on different vesicles, the 100,000×*g* pellet was further separated by a high resolution density gradient designed to better resolve the lower density range typical of sEVs [10]. As expected based on results shown by Jeppesen et al., annexin A1 and A6 were present in sEV fractions with lower densities compared to the exosomal markers Alix, syntenin and Tsg101 (Fig. 6a). CD9 showed a broad distribution through the density gradient, although the fractions containing most of this protein were the same fractions that contained the higher levels of the exosomal markers. This can be explained by the presence of CD9 in sEVs both budding from the plasma membrane and originating from MVBs [62]. Importantly, SNAP29 depletion did not affect the distribution of any of these proteins (Fig. 6b). Furthermore, the distribution of the autophagy-related proteins LC3B and p62 in density gradients was also investigated. Both LC3B and p62 showed a broad distribution with a peak in fraction 4, which has a lower density than fractions were annexin A2 (peak in fraction 6) and syntenin (peak in fraction 8) are found (Fig. 6c). Also in this case, the distribution of LC3B and p62 was not affected by SNAP29 depletion (Fig. 6d). These experiments are in agreement with the idea that the 100,000×*g* pellet contains sEVs of different density and origin and that some members of the annexin protein family and autophagy-related proteins are present on a vesicle population distinct from classical exosomes.

Finally, mass spectrometry was used to investigate whether SNAP29 depletion affects the protein composition of sEVs. In total, 812 proteins were identified in both samples. Only the levels of 17 proteins were significantly different (P ≤ 0.01; fold change ≥ 1.4) between sEVs released by control and SNAP29-depleted cells (Table S3). DOCK10 (also called zizimin3), a member of the DOCK superfamily of Guanine nucleotide Exchange Factor (GEF) proteins, was the protein with the higher fold change. These proteins regulate the Rho family small GTPases and the actin cytoskeleton, cell adhesion and migration [62, 63]. These proteins were included in the Gene Ontology “Cellular compartment” terms: Extracellular exosome, Vesicle, Extracellular region, Plasma membrane and Cell junction. The limited number of proteins and the relatively low fold changes suggests that SNAP29 depletion does not change to a large extent the protein composition of the 100,000×*g* pellet.

### Analysis of SNAP29 cellular localization and MVB morphology and distribution after SNAP29 depletion

**Fig. 7:**
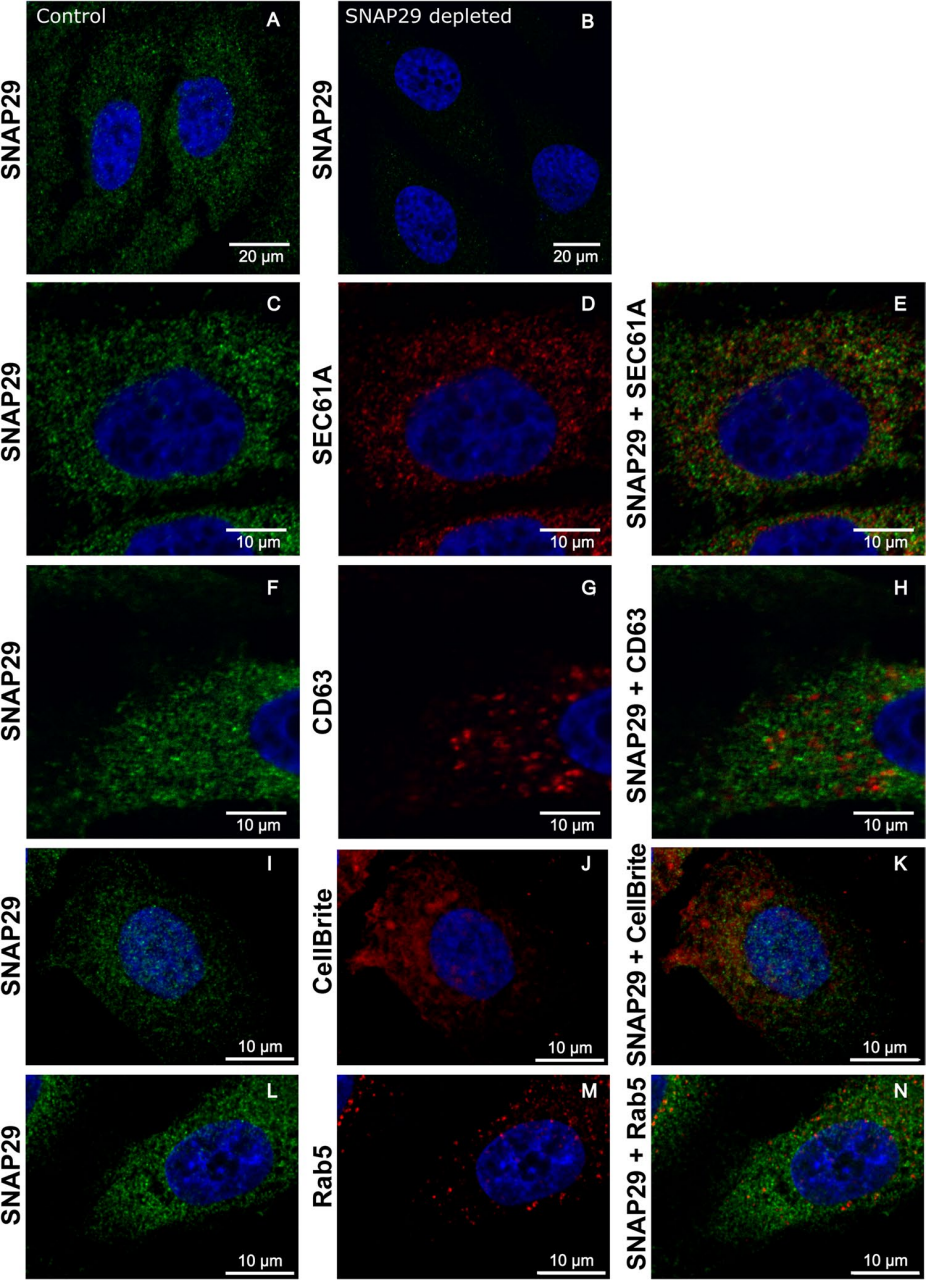
Confocal microscopy analysis of SNAP29 in PC-3 cells. Control PC-3 cells (**A**) and cells depleted of SNAP29 (**B**) were fixed and permeabilized before incubation with SNAP29 antibody and the corresponding secondary antibodies. **C–E** Control cells showing SNAP29 labelling (green), Sec61A (ER) labelling (red), and both proteins together. **F–H** Control cells showing SNAP29 labelling (green), CD63 (MVBs) labelling (red), and both proteins together. **I–K** Control cells showing SNAP29 labelling (green), CellBrite (plasma membrane) staining (red), and a combination of both. **L–N** Control cells showing SNAP29 labelling (green), Rab5 (endosome) labelling (red), and both proteins together. In all cases, cells were washed and mounted with ProLong Gold antifade mounting medium containing DAPI to stain the nuclei (blue) and imaged using a Zeiss LSM710 laser scanning confocal microscope (**A–H**) or a Nikon ECLIPSE Ti2-E confocal spinning disk microscope (**I–N**). Images were captured with a × 63 objective (**A–H**) or a  × 100 objective (**I–N**). Scale bars are indicated (10 μm)

Since our results suggest that the release of exosomes originating from MVBs is affected by SNAP29 depletion, confocal microscopy was performed to investigate the location of SNAP29 and also whether MVBs are affected by SNAP29 depletion. First, the cellular location of SNAP29 in PC-3 cells was investigated and, as shown in Fig. 7a, the protein was found in puncta spread out in the cytoplasm. The signal disappeared in SNAP29 depleted PC-3 cells, thus showing the specificity of the staining (Fig. 7b). A similar cellular distribution of SNAP29 was also observed in the additional cell lines where we investigated sEV release, i.e MCF-7, MDA-MB-231 and Caco-2 cells (Fig.S2).

The cytoplasmic distribution of SNAP29 could resemble that of the endoplasmic reticulum (ER), but the SNAP29 signal did not colocalize with the ER protein Sec61A to a significant extent (Fig. 7c–e). Moreover, SNAP29 was not extensively colocalized with CD63, a protein typically found in MVB, thus indicating that SNAP29 is not found to a large extent in these organelles in PC-3 cells (Fig. 7f–h). Since the presence of SNAP29 at the plasma membrane would support a role in plasma membrane fusion, the potential colocalization of SNAP29 with the plasma membrane was also investigated, but no significant colocalization was observed between SNAP29 and the CellBrite membrane dye (Fig. 7i–k). A similar result was obtained when markers of endosomes (Rab5, EEA1), the Golgi apparatus (GM130) and mitochondria (MitoTracker) were used (Fig. 7l–n, Fig.S3).

**Fig. 8:**
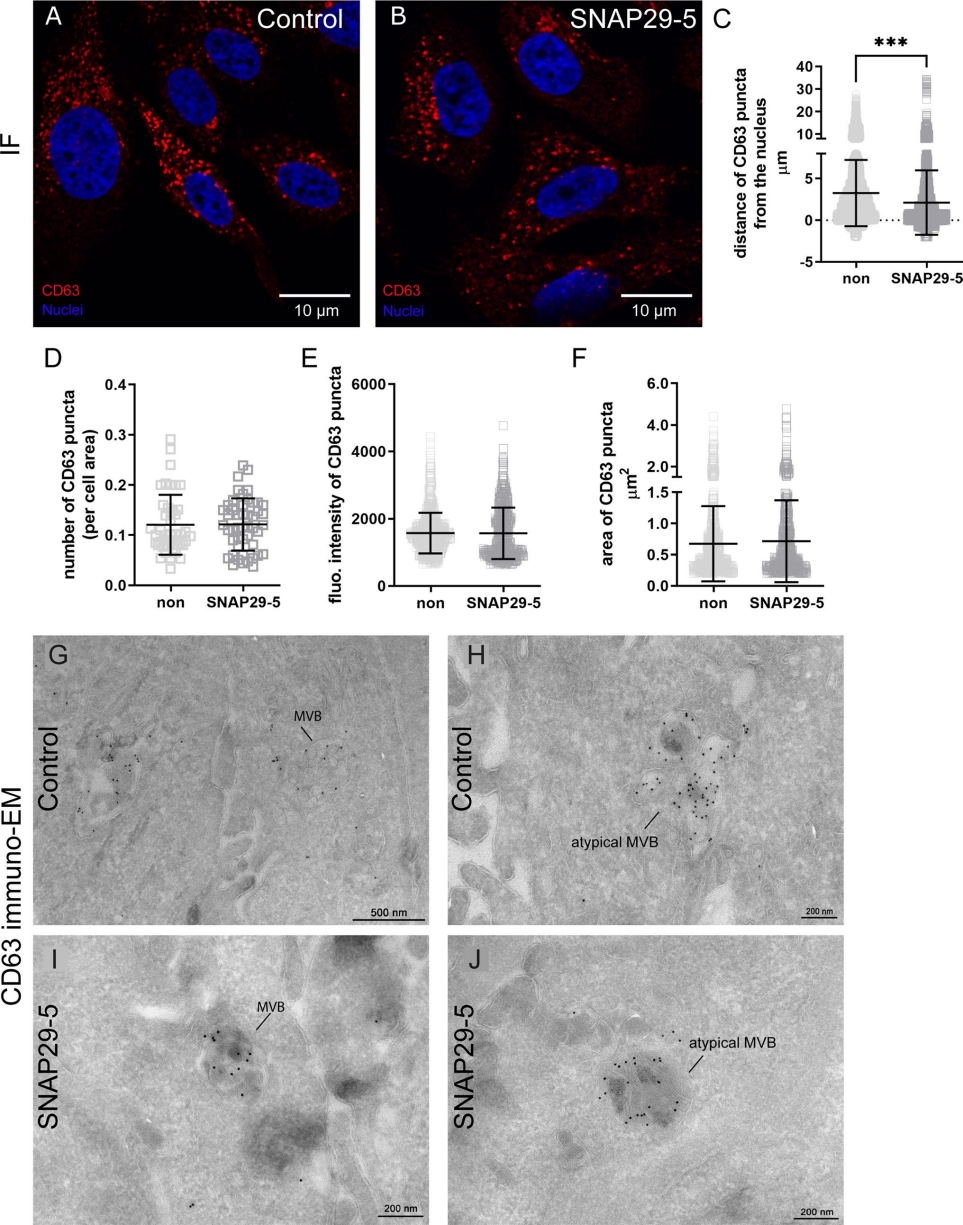
Microscopy analysis of MVBs labelled for CD63 in control and SNAP29 depleted cells. Control PC-3 cells (**A**) and cells depleted of SNAP29 (**B**) were fixed and permeabilized before incubation with CD63 antibody and the corresponding secondary antibodies. Then, cells were washed and mounted with ProLong Gold antifade mounting medium containing DAPI to stain the nuclei (blue) and imaged using a Zeiss LSM710 laser scanning confocal microscope or a Nikon ECLIPSE Ti2-E confocal spinning disk microscope. Z-stack images were captured with a × 63 or × 100 objective. Scale bars are indicated (10 μm). **C** Quantification of the distance of CD63-positive puncta from the nucleus in control (mean = 3.26 nm) and SNAP29 depleted (mean = 2.12 nm) cells. The analysis of Z-stack images was performed by using the function Cell in IMARIS 9.0. At least 120 cells per condition, corresponding to more than 4500 puncta, were analyzed. Data shows mean ± SD. ***P < 0.0001. The quantification of the number of CD63 puncta, normalized to total cell area (**D**), puncta fluorescence intensity (**E**) and puncta area (**F**) was performed on Z-stack projections using ImageJ as described in Materials and Methods in control and SNAP29 depleted PC-3 cells. N ≥ 45 cells per condition or N ≥ 590 puncta per condition. Data shows mean ± SD. Cryo-sectioning and immuno-EM with CD63 (10 nm Au particles) in control cells showing (**G**) a classical MVB morphology and (**H**) an atypical MVB morphology containing multilaminar structures. Cryo-sectioning and immuno-EM with CD63 (10 nm) in SNAP29 depleted cells showing (**I**) classical MVB morphology and (**J**) an atypical MVB morphology containing multilaminar structures

We also investigated the number, morphology and distribution of MVBs after SNAP29 depletion by CD63 labelling (Fig. 8). Image analysis of immunofluorescence data (Fig. 8a, b) showed that the distance of CD63 puncta from the nucleus was higher in control cells (mean = 3.26 nm) compared to SNAP29 depleted cells (mean = 2.12 nm) (Fig. 8c). This could be due to the presence of more acidic CD63-labelled organelles in SNAP29 depleted cells [64, 65]. We could not detect differences in the number of CD63 puncta per cell (Fig. 8d), the fluorescence intensity of CD63 puncta (Fig. 8e) or the area of CD63 puncta (Fig. 8f), suggesting no major alteration of MVB morphology after SNAP29 depletion.

To investigate the ultrastructure of MVBs in more detail, we used cryo-sectioning and immuno-EM. As shown in Fig. 8g–j, PC-3 cells contain CD63-positive MVB compartments that show heterogeneous morphology. Noteworthy, most CD63-positive compartments contained more multilaminar structures than typical ILVs, though some CD63-positive compartments also had classical MVB appearance. However, we could not observe clear differences regarding MVB morphology in control cells (Fig. 8g, h) compared to SNAP29 depleted cells (Fig. 8i, j).

### Analysis of MVB fusion events at the plasma membrane using CD63-pHluorin

**Fig. 9:**
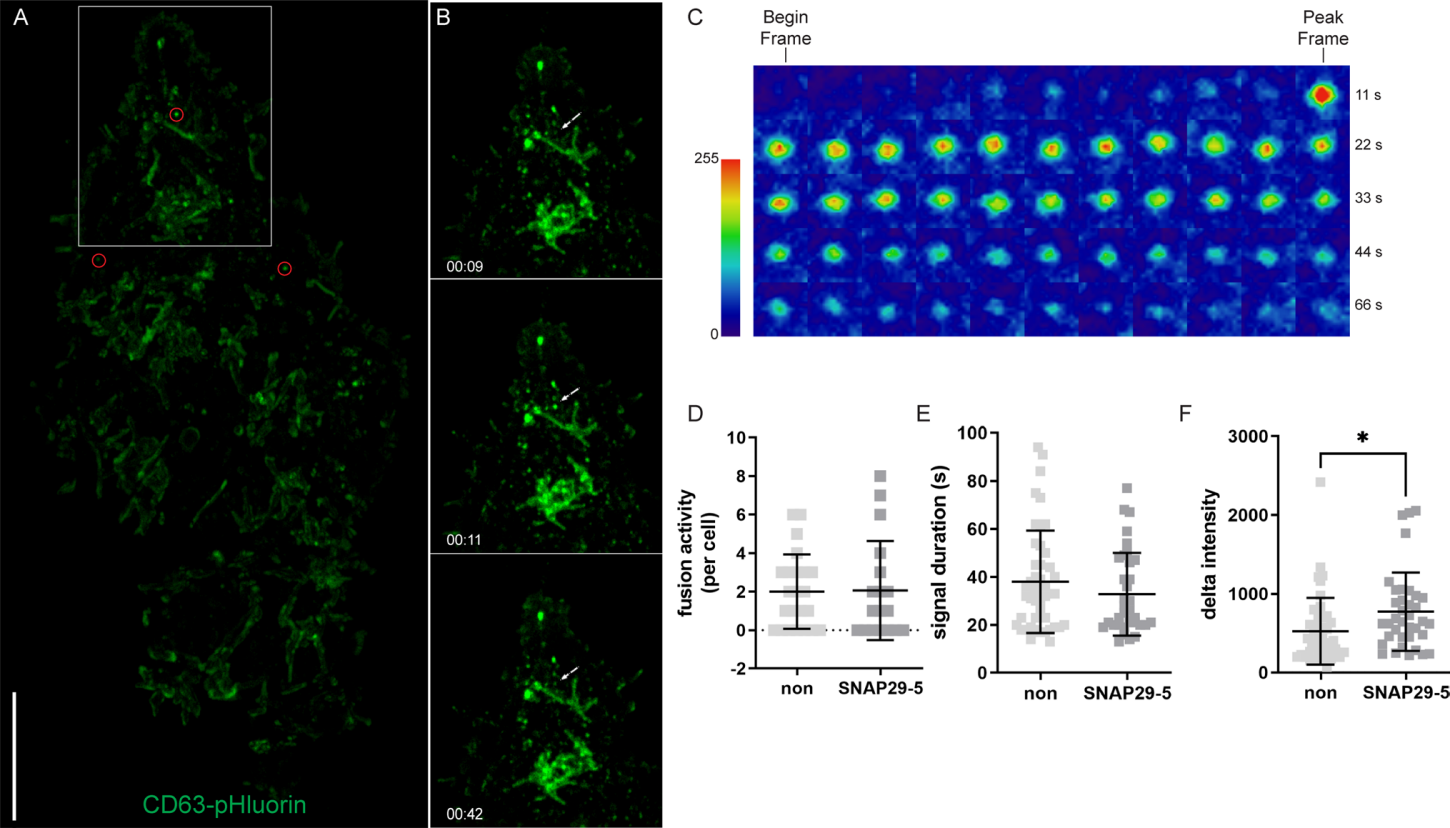
Analysis of MVB fusion events at the plasma membrane using CD63-pHluorin. CD63-pHluorin expressing PC-3 cell were imaged with a Nikon ECLIPSE Ti2-E confocal spinning disk microscope using a × 100 objective at or near the plasma membrane. Image analysis was performed using the ImageJ2/Fiji plugin ExoJ. **A** Total projection of fusion events (red circle) over a time course of 3 min in a representative cell. Scale bar, 10 μm. The white square depicts the region of interest enlarged in **B**. **B** Stills from live imaging of a fusion event (indicated by white arrows) over a time course of 33 s in a representative cell: before the event (top picture), at the start of the event (middle picture), and at the end (bottom picture). **C** Timelapse imaging (heat maps) of a representative CD63-pHluorin fusion event. Begin frame and peak frame are indicated. **D** Quantification of fusion events in control (mean = 2.00) and SNAP29 depleted (mean = 2.06) PC-3 cells transfected with CD63-pHluorin. N ≥ 18 cells per condition. **E** Fluorescent signal duration of CD63 fusion events in control (mean = 38.00 s) and SNAP29 depleted (mean = 32.77 s) cells. N ≥ 37 events per condition. **F** Delta intensity, i.e. the difference of fluorescence intensity between the begin frame and the peak frame, in control (mean = 524.6) and SNAP29 depleted (mean = 772.6) cells. N ≥ 37 events per condition. *P < 0.05; using Student’s two-tailed t test with Welch’s correction. For **D**–**F**, mean ± SD of two independent experiments is shown

In order to get information about the possible step of exosome release in which SNAP29 is involved, PC-3 cells were transiently transfected with CD63-pHluorin, which allows real-time visualization of MVB-plasma membrane fusion [34, 66, 67]. Typical fusions events are shown in Fig. 9a–c and Video1. Quantitative analysis of high-resolution microscopy images of fluorescent signals at or near the plasma membrane showed that the number of fusion events in control (mean = 2.00) and SNAP29 depleted (mean = 2.06) PC-3 cells was similar (Fig. 9d). The fluorescent signal duration of CD63 fusion events in control cell (mean = 38.00 s) compared to SNAP29 depleted cells (mean = 32.77 s) was slightly higher, but the difference was not significant (Fig. 9e). However, we observed that the delta intensity, which represents the difference of intensity between the start of a fusion event (begin frame) and the top signal of the fusion event (peak frame), was higher in SNAP29 depleted cells (mean = 772.6) compared to control cells (mean = 524.6) (Fig. 9f). A possible explanation for this result is that the MVBs that fuse with the plasma membrane in SNAP29 depleted cells are more acidic, which is in agreement with a more juxtanuclear localization of CD63 puncta in these cells (Fig. 8c).

## Discussion

In order to study the role of SNARE proteins in EV secretion in a systematic way, we established a rapid, medium throughput and sensitive screening assay for measuring the release of sEVs based on the incorporation of radioactive cholesterol in vesicles. By using this assay and validating the results by gold standard methods, we found that depletion of the SNARE proteins SNAP29, VAMP8, STX2, STX3 and STX18 decreased sEV release. Importantly, the role of SNAP29 was also validated in three other cancer cell lines: MCF-7, MDA-MB-231 and Caco-2 cells, suggesting a more general role for SNAP29 in sEV secretion.

VAMP7 has previously been described to be involved in exosome secretion in the human leukemic cell line K562 [26, 30]. This SNARE was also identified in our screening, further indicating that the screening assay worked as intended. However, other proteins previously shown to affect exosome release in other cell lines (Rab27, syntenin, PLD2, YKT6, SNAP23 and STX4) were not identified in our screening assay. It should be mentioned that this does not mean that these proteins do not play a role in sEV release, because neither the knockdown efficacy nor the potential toxic effect of the siRNAs were measured in the screening. In order to compensate for these limitations, we used a strict selection parameter: three out of four oligos had to show the same effect. Moreover, it is possible that depletion of some of the proteins could have affected cholesterol synthesis and/or cellular distribution and interfered with the assay. Due to these limitations, the screening could lead to false positive or negative hits. It is therefore essential to confirm the hits identified in the screening using additional independent methods, such as NTA, EM or immunoblotting.

In the present study we found that SNAP29 depletion reduced the number of secreted sEVs as measured both by NTA and EM. It should be noted that the two methods showed a different size distribution of vesicles, with most of the counted particles having a diameter in the 100–175 nm or 30–75 nm range, respectively. Similarly, others have also shown that EV samples measured by NTA give peaks at substantially higher sizes and have broader size distributions as compared to EM [68]. NTA has been shown to correctly estimate the size of standardized particles such as polystyrene and silica beads [69], but EV samples are heterogeneous. Moreover, it has been proposed that EM underestimates the size of vesicles due to shrinking during sample preparation [70], and it is also possible that bigger particles disrupt during this process and/or do not attach well to the grids. Other possible explanation is that EVs may have a protein corona that contributes to their size and that is not detected by EM. The two methods are based on different principles, and this and other factors related to sample preparation probably contribute to the observed differences.

Our results support accumulating evidence showing that the material pelleted at 100,000×*g* is more heterogeneous that previously thought. In addition to exosomes, small ectosomes/microvesicles budding from the plasma membrane as well as material secreted by secretory autophagy and soluble proteins can be co-pelleted during the isolation protocol [9, 10]. It should also be mentioned that exosomes originating from different subtypes of MVBs may also contribute to the heterogeneity of the 100,000×*g* pellet. In terms of small microvesicles/ectosomes, it was recently suggested that annexin A1, A2 and A5 were present on sEVs released from the plasma membrane, but absent from classical exosomes that could be immunoisolated with tetraspanin antibodies [10]. However, these annexins have been localized to endosomes and autophagosomes, and they may to some extent end up in exosomes or in structures secreted via secretory autophagy [71–74].

In the present study we found that it is mainly the release of vesicles containing typical exosomal markers that is affected by SNAP29 depletion. In fact, the amount of some annexins and autophagy-related proteins were unchanged or even slightly increased in sEVs after SNAP29 depletion. Moreover, we found that the 100,000×*g* pellet contains vesicles of slightly different density and protein composition. Importantly, most of annexin A1, A2 and A6 were found in slightly less dense fractions than the classical exosomal markers Alix, Tsg101 and syntenin. In addition, the autophagy-related proteins LC3B and p62 also showed a peak in less dense fractions than the exosomal markers we tested. Finally, it should be mentioned that SNAREs are known to mediate fusion between organelles and between organelles and the plasma membrane, but to our knowledge they have not been reported to be involved in budding of EVs from the plasma membrane. This supports the idea that the effects caused by SNARE proteins in EV release are due to changes in the release of “true” exosomes rather than small ectosomes/microvesicles.

Considering that exosomes originate from MVBs, we also performed microscopy experiments to investigate the cellular localization of SNAP29 and whether the distribution, numbers and/or morphology of MVBs were altered by SNAP29 depletion. SNAP29 is localized in puncta spread out in the cytoplasm that are not clearly associated with specific cellular compartments. We could for example not detect a major colocalization between SNAP29 and MVBs (CD63 labelling) or the plasma membrane (CellBrite dye). However, MVBs were found to be more juxtanuclear in SNAP29 depleted cells compared to control cells. Moreover, experiments with CD63-pHluorin suggest that the CD63-containing organelles that fused with the plasma membrane may be more acidic in SNAP29 depleted cells. In this respect, it has been shown that changes in lysosomal pH are linked to their cellular distribution, and that less acidic lysosomes are often found in the cell periphery while more acidic lysosomes are closer to the nucleus and less prone to be secreted [64, 65]. Moreover, several studies have shown that acidification plays an important role in determining the fate of MVBs, and that less acidic MVBs are more prone to be targeted for secretion [75, 76]. It is therefore possible that SNAP29 depletion could increase endosome acidification, for example by altering the distribution of the vacuolar H^+^ -ATPase, causing a shift in the fate of MVBs towards lysosomes and reducing exosome release.

The percentage of MVBs that fuse with the plasma membrane compared to the ones that fuse with lysosomes is not clearly established. Typically, less than 1% of the CD63 signal in PC-3 cells is associated with sEVs compared to cells when sEV release is measured by immunoblotting [77]. This suggests that only a small percentage of MVBs fuse with the plasma membrane and it may be difficult to detect changes. It should be mentioned that our results did not reveal that the number of MVB fusion events with the plasma membrane or their duration were changed after SNAP29 depletion. However, the method used has some limitations [34, 66], such as the use of transiently transfected PC-3 cells. Moreover, CD63 has been shown to be involved in MVB formation [78], and its overexpression could lead to an increase in exosome release that may mask the effect of SNAP29 depletion. Finally, it is possible that the assay does not capture all the MVB fusion events with the plasma membrane considering that different populations of MVBs may exist [14, 79] and these may have different CD63 levels.

In the present study we found that SNAP29, together with VAMP8, STX2, STX3 and STX18, mediate secretion of sEVs.VAMP7 was found in the screening assay, but was not further validated since its role in exosome release was already known [26, 30]. It is tempting to speculate that SNAP29 acts together with VAMP8 and/or VAMP7 and one or more of the syntaxins STX2, STX3 or STX18 forming a SNARE complex involved in the fusion of MVBs with the plasma membrane. Syntaxins are promiscuous, so it is possible that the SNARE complex can be formed using any of the three identified syntaxins. Moreover, overexpression of SNAP29 has been shown to decrease the presence of VAMP7 at the cell surface [80], and this is expected to impair the exocytic function of VAMP7. It is also possible that different MVB pools utilize slightly different SNARE complexes for fusion with the plasma membrane.

Autophagy/secretory autophagy and exosome secretion share some molecular machinery, and it is known that SNAP29 mediates the fusion of autophagosomes with lysosomes together with STX17 and VAMP8 [81–83]. Interestingly, it has recently been published that both SNAP29 and VAMP8 depletion increase secretory autophagy in the human embryonic kidney HEK-293T cell line [60, 61]. SNAP29 has also been reported to mediate fusion of autophagosomes/amphisomes with the plasma membrane in secretory autophagy together with the SNAREs Sec22b, STX3 and STX4 [58]. Moreover, depletion of SNAP29 has been shown to reduce the extracellular level of a picornavirus (enterovirus D68), which exits the cell through amphisomes [84]. In this study, immunoblotting analysis of the 100,000×*g* fraction did not show significant differences in the level of the autophagy related proteins LC3B and p62 when SNAP29 depleted and control PC-3 cells were compared. Moreover, our proteomic analysis did not show that autophagy related proteins were differentially expressed in the 100,000×*g* fraction when SNAP29 depleted cells were compared to controls cells. These experiments indicate that SNAP29 depletion does not affect secretory autophagy in prostate cancer PC-3 cells. It is not clear why this is the case, but autophagy is regulated by cancer-associated factors [85], and this may cause differences between different cancer cells and/or cancer versus normal cells. Finally, it should be mentioned that SNAP29 has been involved in several trafficking processes in addition to autophagy/secretory autophagy, such as endocytosis, recycling and ER to Golgi transport [86, 87]. We can therefore not exclude that the effect of SNAP29 on sEV release is an indirect one.

In summary, we have established a rapid, medium throughput, and sensitive screening assay for measuring the secretion of sEVs based on the incorporation of radioactive cholesterol in their membranes. By using this assay, we have identified five SNAREs (SNAP29, VAMP8, STX2, STX3 and STX18) involved in sEV secretion. Further studies of SNAP29 suggested that it is specifically the release of exosomes that is affected by SNAP29 depletion. Based on our results, SNARE proteins appear as interesting players in exosome release, and future studies are required to further dissect how they affect the secretion of exosomes and the relationship between different SNAREs in this process. Finally, our results also support the idea that the 100,000×*g* pellet contain a mixture of true exosomes, small microvesicles and material released through secretory autophagy. This has important implications for the interpretation of results based on the isolation of EVs by sequential centrifugation, one of the methods most extensively used by EV researchers.

### Supplementary Information

Below is the link to the electronic supplementary material.

Supplementary file1 (PDF 786 KB)

Supplementary file2 Video 1. Time-lapse imaging of a representative CD63-pHluorin PC-3 cell at 5× normal speed. Shot at five frames per second. White circles indicate fusion events. Scale bar, 10 μm. (MP4 6842 KB)

## Data Availability

The datasets generated during the current study are available under request or in the PRIDE repository, with the dataset identifierPXD040958.

## Abbreviations

EM: Electron microscopy
ESCRTs: Endosomal sorting complexes required for transport
EV: Extracellular vesicle
ILV: Intraluminal vesicle
kd: Knockdown
MS: Mass spectrometry
MVB: Multivesicular body
nLC-MS/MS: Nano liquid chromatography-tandem mass spectrometry
NTA: Nanoparticle tracking analysis
sEV: Small extracellular vesicles
SNAP: Synaptosomal-associated protein
SNARE: Soluble N-ethylmaleimide-sensitive factor attachment protein receptors
STX: Syntaxin
VAMP: Vesicle-associated membrane protein
