# Supplementary material for: siRNA screening reveals that SNAP29 contributes to exosome release
Source: Cell Mol Life Sci. 2023 Jun 7;80(7):177. doi: 10.1007/s00018-023-04822-8 (PMC10247572; doi:10.1007/s00018-023-04822-8)

SUPPLEMENTARY INFORMATION

Supplementary table legends

Table S1. List of proteins included in the siRNA screen.
Table S2. Sequences for all siRNAs included in the siRNA screen.
Table S3. Proteins that were affected ($p \leq 0.01$; fold change$\geq$1.4) in sEVs released from cells depleted of SNAP29 compared to control cells. Blue color indicates downregulated proteins, pink color indicates upregulated proteins; FDR$\leq$0.1. Four independent analysis were performed.

Supplementary figure legends

Fig. S1. Size distribution of sEVs shown as number of particles normalized by total number of particles for each condition. PC-3 cells were depleted for STX2 (A), STX3 (B), STX18 (C), VAMP8 (D), and (E) SNAP29, before sEVs were isolated by sequential centrifugation and their size was measured by NTA. The data shows mean + SEM from 3-4 independent experiments.

Fig. S2. Confocal microscopy analysis of SNAP29 in MCF7, MDA-MB-231 and Caco2 cell. SNAP29 labelling (green) in (A) MCF7, (B) MDA-MB-231 and (C) Caco2 cells. In all cases, cells were fixed and permeabilized before incubation with SNAP29 antibody and the corresponding secondary antibodies. Cells were then washed and mounted with ProLong Gold antifade mounting medium containing DAPI to stain the nuclei (blue) and imaged using a Nikon ECLIPSE Ti2-E confocal spinning disk microscope. Images were captured with a x100 objective. Scale bars are indicated (10 μm).

Fig. S3. Confocal microscopy analysis of SNAP29 in control PC-3 cells. (A-C) Cells showing SNAP29 labelling (green), EEA1 (early endosome) labelling (red), and both proteins together. (D-F) Cells showing SNAP29 labelling (green), GM130 (Golgi apparatus) labelling (red), and both proteins together. (G-H) Control cells showing SNAP29 labelling (green), MitoTracker (mitochondria) staining (red), and a combination of both. In all cases, cells were washed and mounted with ProLong Gold antifade mounting medium containing DAPI to stain the nuclei (blue) and imaged using a Nikon ECLIPSE Ti2-E confocal spinning disk microscope. Images were captured with a x100 objective. Scale bars are indicated (10 μm).

Table S1. List of proteins included in the siRNA screen.

Category	Gene Symbol	UniprotKB name
**SNARE and SNARE-associated proteins**	YKT6	Synaptobrevin homolog YKT6
	SNAP23	Synaptosomal-associated protein 23
	SNAP25	Synaptosomal-associated protein 25
	SNAP29	Synaptosomal-associated protein 29
	BNIP1	Vesicle transport protein SEC20
	SEC22A	Vesicle-trafficking protein SEC22a
	SEC22B	Vesicle-trafficking protein SEC22b
	SEC22C	Vesicle-trafficking protein SEC22c
	VAMP7	Vesicle-associated membrane protein 7
	VAMP8	Vesicle-associated membrane protein 8
	STX2	Syntaxin-2
	STX3	Syntaxin-3
	STX4	Syntaxin-4
	STX7	Syntaxin-7
	STX16	Syntaxin-16
	STX18	Syntaxin-18
	STXBP3	Syntaxin-binding protein 3
	USE1	Vesicle transport protein USE1
	VTI1B	Vesicle transport through interaction with t-SNAREs homolog 1B
	GOSR1	Golgi SNAP receptor complex member 1
	GOSR2	Golgi SNAP receptor complex member 2
	BET1	BET1 homolog
	BET1L	BET1-like protein
	SYT1	Synaptotagmin-1
	SYTL2	Synaptotagmin-like protein 2
**Ras superfamily of small GTPases**	RAB27A	Ras-related protein Rab-27A
	RAB27B	Ras-related protein Rab-27B
	CDC42	Cell division control protein 42 homolog
	RHOD	Rho-related GTP-binding protein RhoD
	RHOF	Rho-related GTP-binding protein RhoF
	RND3	Rho-related GTP-binding protein RhoE
	ARL13B	ADP-ribosylation factor-like protein 13B
	ARL15	ADP-ribosylation factor-like protein 15
**Phospholipases**	PLCB4	1-phosphatidylinositol 4,5-bisphosphate phosphodiesterase beta-4
	PLCD1	1-phosphatidylinositol 4,5-bisphosphate phosphodiesterase delta-1
	PLCD3	1-phosphatidylinositol 4,5-bisphosphate phosphodiesterase delta-3
	PLD1	Phospholipase D1
	PLD2	Phospholipase D2
**Tight junctions**	CLDN1	Claudin-1
	CLDN3	Claudin-2
	CLDN4	Claudin-3
**Phospholipid scramblases**	PLSCR1	Phospholipid scramblase 1
	PLSCR3	Phospholipid scramblase 3
**Sortin nexins**	SNX11	Sorting nexin-11
	SNX17	Sorting nexin-17
**Others**	GRASP	GRP1-associated scaffold protein
	GORASP1	Golgi reassembly-stacking protein 1
	GORASP2	Golgi reassembly-stacking protein 2
	SDCBP	Syntenin-1

Table S2. Sequences for all siRNAs included in the siRNA screen.

Duplex Catalog Number	Gene Symbol	GENE ID	Gene Accession	GI Number	Sequence
J-004667-05	RAB27A	5873	NM_183236	34485710	CCAUAGCACUCGCAGAGAA
J-004667-06	RAB27A	5873	NM_183236	34485710	CAGGAGAGGUUUCGUAGCU
J-004667-07	RAB27A	5873	NM_183236	34485710	UCACAACAGUGGGCAUUGA
J-004667-08	RAB27A	5873	NM_183236	34485710	CUACGGAUCAGUUAAGUGA
J-004228-05	RAB27B	5874	NM_004163	21314628	GGAACUGGCUGACAAAUAU
J-004228-06	RAB27B	5874	NM_004163	21314628	GCAAAUGCUUAUUGUGAAA
J-004228-07	RAB27B	5874	NM_004163	21314628	GGAAGUCAAUGAACGGCAA
J-004228-08	RAB27B	5874	NM_004163	21314628	UGAAACAAGUGCAGCAACU
J-019237-09	YKT6	10652	NM_006555	34304384	GCUCAAAGCCGCAUACGAU
J-019237-10	YKT6	10652	NM_006555	34304384	CUAUAAAACUGCCCGGAAA
J-019237-11	YKT6	10652	NM_006555	34304384	AUACCAGAACCCACGAGAA
J-019237-12	YKT6	10652	NM_006555	34304384	CUAAAGUGCAGGCCGAACU
J-011959-06	GOSR1	9527	NM_001007025	55770857	CAGAAGAACUGAGCUAUUU
J-011959-07	GOSR1	9527	NM_001007025	55770857	GAAGUCAAUUCACAGCAAA
J-011959-08	GOSR1	9527	NM_001007025	55770857	GGGAGAAUCUUAUGGGAUC
J-011959-09	GOSR1	9527	NM_001007025	55770857	GAGACAUAUUGCAGGAUUA
J-010980-06	GOSR2	9570	NM_054022	60499002	ACGAAUCACUGCAGUUUAA
J-010980-07	GOSR2	9570	NM_054022	60499002	CGAAAUCCAAGCAAGCAUA
J-010980-08	GOSR2	9570	NM_054022	60499002	GAUCCAGUCUUGCAUGGGA
J-010980-09	GOSR2	9570	NM_054022	60499002	UUAGAUGGGCACAAUAUUU
J-017545-05	SNAP23	8773	NM_130798	18765730	UAACAGAACUCAACAAAUG
J-017545-06	SNAP23	8773	NM_130798	18765730	CAACUAAACCGCAUAGAAG
J-017545-07	SNAP23	8773	NM_130798	18765730	GAAACUCAUUGACAGCUAA
J-017545-08	SNAP23	8773	NM_130798	18765730	ACAGAGAUCGUAUUGAUAU
J-011394-09	SNAP25	6616	NM_130811	18765734	CUGGAAAGCACCCGUCGUA
J-011394-10	SNAP25	6616	NM_130811	18765734	CAGAAUCGCCAGAUCGACA
J-011394-11	SNAP25	6616	NM_130811	18765734	GUGUAGUGGACGAACGGGA
J-011394-12	SNAP25	6616	NM_130811	18765734	ACAAAUGAUGCCCGAGAAA
J-011935-05	SNAP29	9342	NM_004782	18765736	GAAGCUAUAAGUACAAGUA
J-011935-06	SNAP29	9342	NM_004782	18765736	GACAAGAUGGACCAAGAUU
J-011935-07	SNAP29	9342	NM_004782	18765736	CAACCAAAGUGGACAAGUU
J-011935-08	SNAP29	9342	NM_004782	18765736	UUAGAAAGCUGGAUGAUAC
J-012479-09	SEC22A	26984	NM_012430	14591918	GGGCAAGGCUCCCGAUUAU
J-012479-10	SEC22A	26984	NM_012430	14591918	AGGACCAAGCAGCGAUAUA
J-012479-11	SEC22A	26984	NM_012430	14591918	GAACAUCACAAGUAUCGAA
J-012479-12	SEC22A	26984	NM_012430	14591918	CAAAUUUCCAUGUGCGAAC
J-011963-05	SEC22B	9554	NM_004892	34335289	AAUAGUGUAUGUCCGAUUC
J-011963-06	SEC22B	9554	NM_004892	34335289	GCUAAGCAACUCUUUCGAA
J-011963-07	SEC22B	9554	NM_004892	34335289	CCUAGAAGAUUUGCACUCA
J-011963-08	SEC22B	9554	NM_004892	34335289	GAAGCACUCUCAGCAUUGG
J-011911-09	SEC22C	9117	NM_032970	21536309	CCUCCAGGCCAUACGCUUU
J-011911-10	SEC22C	9117	NM_032970	21536309	CCGAAUGGAACCAGUGACA
J-011911-11	SEC22C	9117	NM_032970	21536309	GGUUGUGACUUUAGUAUAC
J-011911-12	SEC22C	9117	NM_032970	21536309	GUAUCCAGGUCGAGGUUCU
J-020864-05	VAMP7	6845	NM_005638	27545446	GUACUCACAUGGCAAUUAU
J-020864-06	VAMP7	6845	NM_005638	27545446	GAACGUUCCCGAGCCUUUA
J-020864-07	VAMP7	6845	NM_005638	27545446	CGAGUUCUCAAGUGUCUUA
J-020864-08	VAMP7	6845	NM_005638	27545446	GCCAAGACAGGAUUGUAUA
J-013503-05	VAMP8	8673	NM_003761	14043025	CCACUGGUGCCUUCUCUUA
J-013503-06	VAMP8	8673	NM_003761	14043025	GUCCUUAUCUGCGUGAUUG
J-013503-07	VAMP8	8673	NM_003761	14043025	GAAAUGAUCGUGUGCGGAA
J-013503-08	VAMP8	8673	NM_003761	14043025	GGGAAAACUUGGAACAUCU
J-021379-05	STX2	2054	NM_001980	37577286	UAGACAAGCUCUCAAUGAA
J-021379-06	STX2	2054	NM_001980	37577286	CUUGUGAUCCUUGGAAUUA
J-021379-07	STX2	2054	NM_001980	37577286	UAGCAACCAUAUCCCAAGA
J-021379-08	STX2	2054	NM_001980	37577286	GUUAAAGGCUAUUGAACAA

J-015401-05	STX3	6809	NM_004177	34147491	GAUCAUUGACUCACAGAUU
J-015401-06	STX3	6809	NM_004177	34147491	AAGAAACUCUACAGUAUCA
J-015401-07	STX3	6809	NM_004177	34147491	AGGGUGAGAUGUUAGAUAA
J-015401-08	STX3	6809	NM_004177	34147491	AAACUCGGCUUAACAUUGA
J-016256-05	STX4	6810	NM_004604	34147603	GGACAAUUCGGCAGACUAU
J-016256-06	STX4	6810	NM_004604	34147603	GCGUCACAGUGGUUGGAUA
J-016256-07	STX4	6810	NM_004604	34147603	GCGAGGUGUUUGUGUCCAA
J-016256-08	STX4	6810	NM_004604	34147603	CGUCAACACAAGAAUGAGA
J-019551-09	STX7	8417	NM_003569	4507294	GAGUUUGUUGCUCGAGUAA
J-019551-10	STX7	8417	NM_003569	4507294	CAGCAGAUUAUCAGCGCAA
J-019551-11	STX7	8417	NM_003569	4507294	UGUCAUUGGAGUUGCGAUU
J-019551-12	STX7	8417	NM_003569	4507294	GGAGCACACUGUCGCACUA
J-019468-09	STX16	8675	NM_001001434	47778944	GUAUGAUGUUGGCCGGAUU
J-019468-10	STX16	8675	NM_001001434	47778944	AAAUAUUCCACCAGCGAUU
J-019468-11	STX16	8675	NM_001001434	47778944	GCUAAUUGAGAGAGCGUUA
J-019468-12	STX16	8675	NM_001001434	47778944	AGUAGGACUUCAUCGUUUA
J-020624-09	STX18	53407	NM_016930	39725935	CUGUCUGAGUCAACGAAUA
J-020624-10	STX18	53407	NM_016930	39725935	CAGAGAGCCAUCCGAGUUA
J-020624-11	STX18	53407	NM_016930	39725935	GUUCAGACAGACAGACGUA
J-020624-12	STX18	53407	NM_016930	39725935	CAAGAGAUAUUCACGGAAA
J-021121-09	USE1	55850	NM_018467	49574516	UGAAGGUCCACGCGAGCAA
J-021121-10	USE1	55850	NM_018467	49574516	GAGCCUGAGAUGGACGUAA
J-021121-11	USE1	55850	NM_018467	49574516	AGCUAAACCUGGUGCGGCU
J-021121-12	USE1	55850	NM_018467	49574516	CGAAUCAUGCCUAAACUCA
J-020152-09	VTI1B	10490	NM_006370	5454165	CCAAAGUAUUGAACGUUCU
J-020152-10	VTI1B	10490	NM_006370	5454165	GAGCAUAUGAAUCGGCUAC
J-020152-11	VTI1B	10490	NM_006370	5454165	UGGAGGAGGAGCUACGUUA
J-020152-12	VTI1B	10490	NM_006370	5454165	UUGCUAAACUCCAUCGGGA
J-012085-09	BET1	10282	NM_005868	83779007	GGAACUAUGGCUAUGCUAA
J-012085-10	BET1	10282	NM_005868	83779007	GAAACUUAGUGGGAGAGUA
J-012085-11	BET1	10282	NM_005868	83779007	AAAUAGGCCAUGAAGUUAA
J-012085-12	BET1	10282	NM_005868	83779007	CCUAAUAACCUGUCAGUUU
J-018850-09	BET1L	51272	NM_016526	34365798	UCCCUUAGGACUCGGAUUU
J-018850-10	BET1L	51272	NM_016526	34365798	GCGGGUGGGUGGUCAAAUC
J-018850-11	BET1L	51272	NM_016526	34365798	AGUGUGAGAGCCAGGGUUA
J-018850-12	BET1L	51272	NM_016526	34365798	CCUUCUAGCUUGCUGGUAA
J-011222-05	BNIP1	662	NM_013978	153946402	GGUCCUCUAUAUUGUGAAA
J-011222-06	BNIP1	662	NM_013978	153946402	ACAGUUGCGUCACAGAAUA
J-011222-08	BNIP1	662	NM_013978	153946402	GGAUAUCCGUGAUUGUUCA
J-011222-18	BNIP1	662	NM_013978	153946402	UCUCAUCUUCCUUGCGCUA
J-018892-05	GRASP	160622	NM_181711	32171220	GGAGAGUACAGGUCCCUAA
J-018892-06	GRASP	160622	NM_181711	32171220	CAUCCGGCAUCGAGAGAUU
J-018892-07	GRASP	160622	NM_181711	32171220	UAACCAGACCUUCGGCUUU
J-018892-08	GRASP	160622	NM_181711	32171220	CAGCGUCAAUGGCCUGAAU
J-005057-05	CDC42	998	NM_044472	16357471	CGGAAUAUGUACCGACUGU
J-005057-06	CDC42	998	NM_044472	16357471	GCAGUCACAGUUAUGAUUG
J-005057-07	CDC42	998	NM_044472	16357471	GAUGACCCCUCUACUAUUG
J-005057-08	CDC42	998	NM_044472	16357471	CUGCAGGGCAAGAGGAUUA
J-013510-05	GORASP1	64689	NM_031899	29826292	GAUCUCUACCACAGAAUAA
J-013510-06	GORASP1	64689	NM_031899	29826292	GAGGACUUCUUUACGCUCA
J-013510-07	GORASP1	64689	NM_031899	29826292	GAACUGACCACCACAGCUG
J-013510-08	GORASP1	64689	NM_031899	29826292	CUGGAGGUGUUCAAUAUGA
J-019045-05	GORASP2	26003	NM_015530	29826293	CCACACAGUGAUUAUAUAA
J-019045-06	GORASP2	26003	NM_015530	29826293	GCAGAUACAGUCAUGAAUG
J-019045-07	GORASP2	26003	NM_015530	29826293	CAUUGGAUAUGGUUAUUUG
J-019045-08	GORASP2	26003	NM_015530	29826293	GGAGUGAGCAUUCGUUUCU

J-017365-09	ARL13B	200894	NM_144996	33598957	CUUUAAGUGUACAUCGUAA
J-017365-10	ARL13B	200894	NM_144996	33598957	UGAACGAGUGCGAAAAUUA
J-017365-11	ARL13B	200894	NM_144996	33598957	AGACACAAGGCCAGGUUAA
J-017365-12	ARL13B	200894	NM_144996	33598957	GCUACAUGUUAUUGCAAGA
J-020355-09	ARL15	54622	NM_019087	9506652	CCGAAUAACUGAGGCGUUU
J-020355-10	ARL15	54622	NM_019087	9506652	GCUGAUAACAUCCGGAAAU
J-020355-11	ARL15	54622	NM_019087	9506652	CCACAGACUCACACUAUAU
J-020355-12	ARL15	54622	NM_019087	9506652	GCAUUAACGUCAAGUACAC
J-009594-05	PLCB4	5332	NM_182797	33469938	GGUCAGAAAUGAUCAAUAC
J-009594-06	PLCB4	5332	NM_182797	33469938	GAUCAAUCAUACACAACUU
J-009594-07	PLCB4	5332	NM_182797	33469938	CCACUGACACCAUACGUAA
J-009594-08	PLCB4	5332	NM_182797	33469938	CAAGGAAACUGCAUUUGUC
J-009149-05	PLCD1	5333	NM_006225	195972884	GGAGUUUGCGUUUGAGGUA
J-009149-07	PLCD1	5333	NM_006225	195972884	UAUGAUGCCUCCUCCAAGA
J-009149-08	PLCD1	5333	NM_006225	195972884	GGACCAGCGUCAGAAGCUA
J-009149-18	PLCD1	5333	NM_006225	195972884	CAAUCAUCUACCACGGCUA
J-008649-09	PLCD3	113026	NM_133373	48675819	UGAACGACAUGUACGCCUA
J-008649-10	PLCD3	113026	NM_133373	48675819	AAGCAAGGGUACCGCCACA
J-008649-11	PLCD3	113026	NM_133373	48675819	CCUAUGAGCUCAACGAGAC
J-008649-12	PLCD3	113026	NM_133373	48675819	GACAGUAGAAUGAGCCUAU
J-017369-09	CLDN1	9076	NM_021101	21536297	GCAAUAGAAUCGUUCAAGA
J-017369-10	CLDN1	9076	NM_021101	21536297	GGAAAGACUACGUGUGACA
J-017369-11	CLDN1	9076	NM_021101	21536297	CACCAAGGCCCUAUCCAAA
J-017369-12	CLDN1	9076	NM_021101	21536297	GAGGAUGGCUGUCAUUGGG
J-017303-05	CLDN3	1365	NM_001306	21536298	CCACCAAGGUCGUCUACUC
J-017303-06	CLDN3	1365	NM_001306	21536298	GGACUACGUCUAAGGGACA
J-017303-07	CLDN3	1365	NM_001306	21536298	GCAGCAACAUCAUCACGUC
J-017303-08	CLDN3	1365	NM_001306	21536298	CAUCGUGUGCUGCGCGUUG
J-013612-05	CLDN4	1364	NM_001305	34335232	GCAACUACGUGUAAGGUGC
J-013612-06	CLDN4	1364	NM_001305	34335232	ACAAUCCGCUGGUGGCCUC
J-013612-07	CLDN4	1364	NM_001305	34335232	CCCACAACAUCAUCCAAGA
J-013612-08	CLDN4	1364	NM_001305	34335232	GGUAAUGGGCAUCGCGCUG
J-009413-05	PLD1	5337	NM_002662	46276864	CAACAGAGUUUCUUGAUAU
J-009413-06	PLD1	5337	NM_002662	46276864	GGUAAUCAGUGGAUAAAUU
J-009413-07	PLD1	5337	NM_002662	46276864	CCAUGGAGGUUUGGACUUA
J-009413-08	PLD1	5337	NM_002662	46276864	CCGGGUAUAUGUCGUGAUA
J-005064-05	PLD2	5338	NM_002663	20070140	GGACCGGCCUUUCGAAGAU
J-005064-06	PLD2	5338	NM_002663	20070140	CAGCAUGGCGGGACUAUAU
J-005064-07	PLD2	5338	NM_002663	20070140	CAAGGUGGGCGAUGAGAUU
J-005064-08	PLD2	5338	NM_002663	20070140	ACAUUAUGCUCAAGAGGAA
J-003729-07	PLSCR1	5359	NM_021105	10863876	UUACAGACGCUGAUAACUU
J-003729-08	PLSCR1	5359	NM_021105	10863876	GAUAAUAUGGGUCAAGAAG
J-003729-09	PLSCR1	5359	NM_021105	10863876	CCAAAUCAGCCAGUGUAUA
J-003729-10	PLSCR1	5359	NM_021105	10863876	GUAGUGGAUUAGUGAAAGU
J-010255-05	PLSCR3	57048	NM_020360	31543416	GGAGACCUGUAAUCGGUAU
J-010255-06	PLSCR3	57048	NM_020360	31543416	GCAGAUGACUUUGGCCUAC
J-010255-07	PLSCR3	57048	NM_020360	31543416	CCACAUUCCUCAUUGACUA
J-010255-08	PLSCR3	57048	NM_020360	31543416	GGUGAAGACUCGGGAUGAA
J-008940-05	RHOD	29984	NM_014578	17738304	CGGCUCGGCUCCAUGACAA
J-008940-06	RHOD	29984	NM_014578	17738304	CCACGGUGUUUGAGCGGUA
J-008940-07	RHOD	29984	NM_014578	17738304	GCAAGAAGGUACCCAUCAU
J-008940-08	RHOD	29984	NM_014578	17738304	GAUUGGAGCCUGUGACCUA

J-007794-06	RND3	390	NM_005168	56676394	CUACAGUGUUUGAGAAUUA
J-007794-07	RND3	390	NM_005168	56676394	UAGUAGAGCUCUCCAAUCA
J-007794-08	RND3	390	NM_005168	56676394	CAGCAAAUCUAUCAUGGAU
J-007794-09	RND3	390	NM_005168	56676394	GCGGACAGAUGUUAGUACA
J-008316-05	RHOF	54509	NM_019034	38016956	CAUCGGUGUUCGAGAAGUA
J-008316-06	RHOF	54509	NM_019034	38016956	GCAAGACAGACCUGAGGAA
J-008316-07	RHOF	54509	NM_019034	38016956	UCCCUGAGGUCACGCAUUU
J-008316-08	RHOF	54509	NM_019034	38016956	UGAAGAAGGCGCAACGGCA
J-013673-09	SNX11	29916	NM_013323	23111025	GGUGUAGGAUGUCGGAGAA
J-013673-10	SNX11	29916	NM_013323	23111025	CAUUGAGAAGCGACGACAA
J-013673-11	SNX11	29916	NM_013323	23111025	GGAUUUAUGUCCCGAGACC
J-013673-12	SNX11	29916	NM_013323	23111025	GUAUGUAGGACCAGGAGUA
J-013427-09	SNX17	9784	NM_014748	23238249	CAUGCAAGCUGUUCGGCAA
J-013427-10	SNX17	9784	NM_014748	23238249	AUGCGAUGCUGGCGGGUCA
J-013427-11	SNX17	9784	NM_014748	23238249	CUUUAUGCUCAGACGGUAU
J-013427-12	SNX17	9784	NM_014748	23238249	CCAGUGAUGUCCACGGCAA
J-020044-05	SYT1	6857	NM_005639	5032138	GCAAUUUACUUUCAAGGUA
J-020044-06	SYT1	6857	NM_005639	5032138	GGGCACAUCUGAUCCUUAC
J-020044-07	SYT1	6857	NM_005639	5032138	GAUCGUUUCUCUAAGCAUG
J-020044-08	SYT1	6857	NM_005639	5032138	GUAAGAGGCUGAAGAAGAA
J-013555-05	SYTL2	54843	NM_032379	46255060	GAAACUAGCUCUCCAGUAU
J-013555-06	SYTL2	54843	NM_032379	46255060	CAUUUAAGCGCAAUAGUUU
J-013555-07	SYTL2	54843	NM_032379	46255060	UGUAAAGGCCUAUUUGCUA
J-013555-08	SYTL2	54843	NM_032379	46255060	GGACUGGGAUAACAAACAG
J-019626-09	STXBP3	6814	NM_007269	6005885	AGAGUGACAUGAUUCGUAA
J-019626-10	STXBP3	6814	NM_007269	6005885	UCAACAAGGCAAACCGUUA
J-019626-11	STXBP3	6814	NM_007269	6005885	GUAAAUCGGAGAACAAGUA
J-019626-12	STXBP3	6814	NM_007269	6005885	GAAGAUGACCUCUGGGUUA
J-008270-05	SDCBP	6386	NM_001007068	55749503	GGAGAGAAGAUUACCAUGA
J-008270-06	SDCBP	6386	NM_001007068	55749503	GACCAAGUACUUCAGAUCA
J-008270-07	SDCBP	6386	NM_001007068	55749503	GGAUGGUCUUAGAAUAUUU
J-008270-08	SDCBP	6386	NM_001007068	55749503	GCAUUUGACUCUUAAGAUU

Table S3. Proteins affected (p≤0.01; fold change≥1.4) in small EVs released from SNAP29 depleted compared to control cells.

Protein names	Gene names	p-value	Fold change SNAP29/control
Dedicator of cytokinesis protein 10	DOCK10	0,00015	-1,69
Anion exchange protein	SLC4A7	0,00033	1,5
Choline transporter-like protein 2	SLC44A2	0,00138	-1,71
Podocalyxin	PODXL	0,00144	-1,78
1-phosphatidylinositol 4,5-bisphosphate phosphodiesterase beta-4	PLCB4	0,00186	1,43
Anoctamin-1	ANO1	0,00203	-1,7
Thioredoxin	TXN	0,00227	1,46
Adenylyl cyclase-associated protein 1	CAP1	0,00344	-1,4
Guanine nucleotide-binding protein subunit alpha-11	GNA11	0,00362	1,43
Annexin A1	ANXA1	0,00409	1,49
G-protein coupled receptor family C group 5 member C	GPRC5C	0,00478	-1,89
Na(+)/H(+) exchange regulatory cofactor NHE-RF1	SLC9A3R1	0,00539	-1,44
Ras-related protein R-Ras2	RRAS2	0,00684	-1,5
Alkaline phosphatase	ALPP	0,00690	-1,5
F-actin-capping protein subunit beta	CAPZB	0,00795	-1,64
Hyaluronan synthase 3	HAS3	0,00866	1,78
Tyrosine-protein kinase transmembrane receptor ROR1	ROR1	0,00881	-1,76

Supplementary figure 1

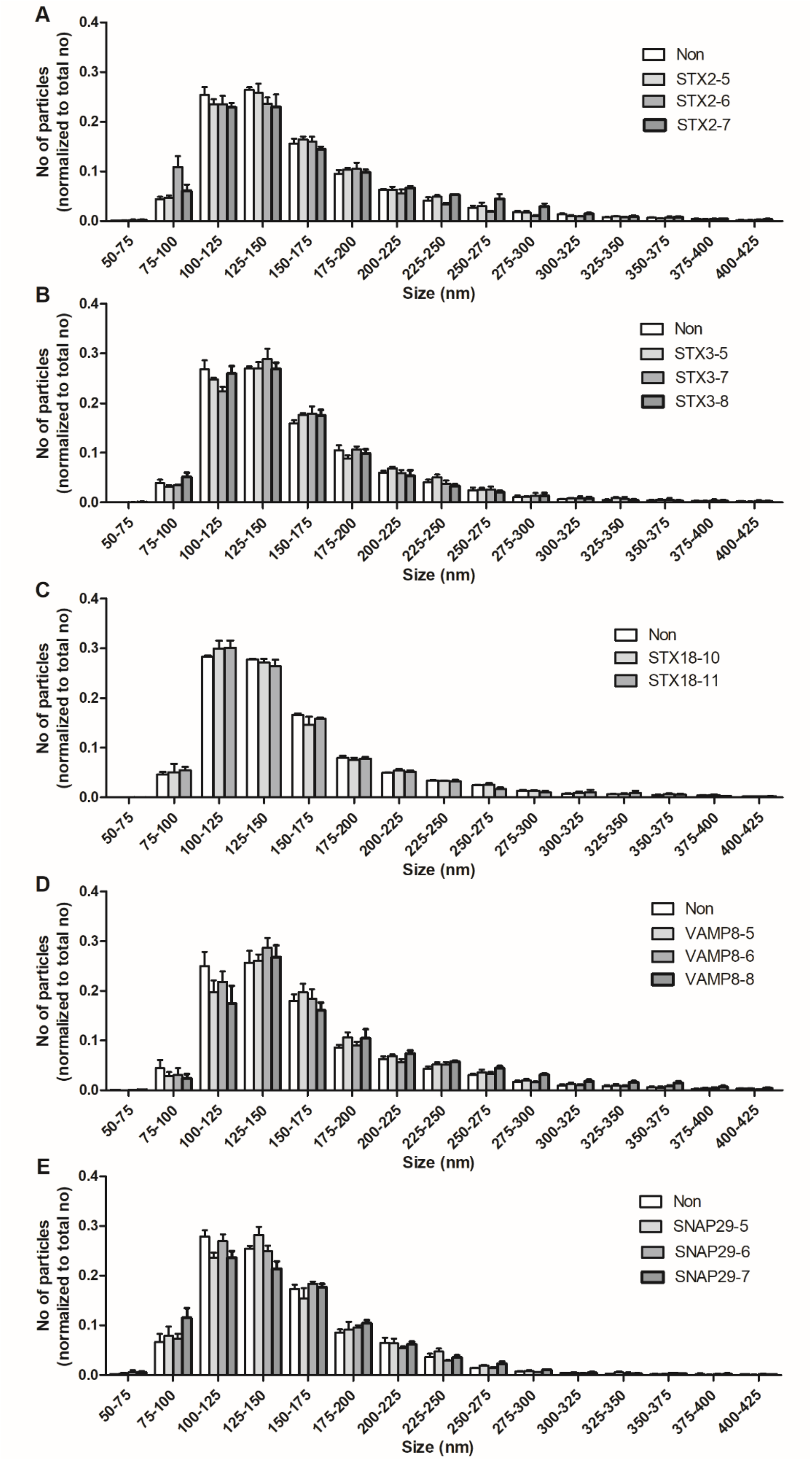

Supplementary figure 2

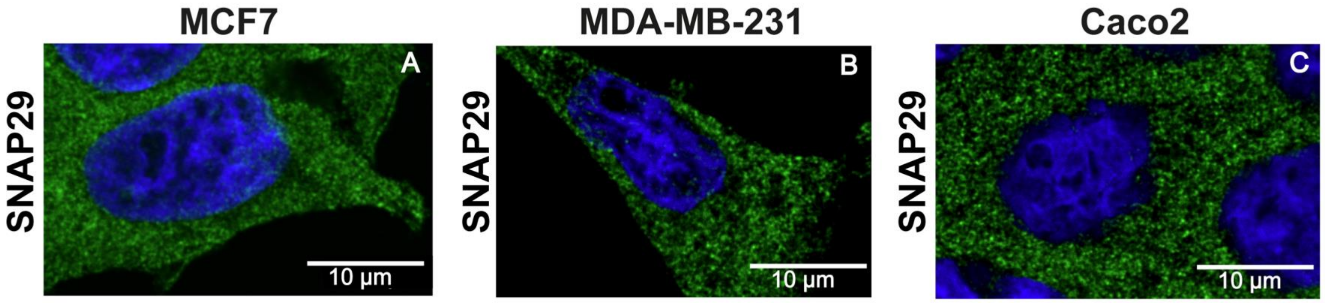

Supplementary figure 3

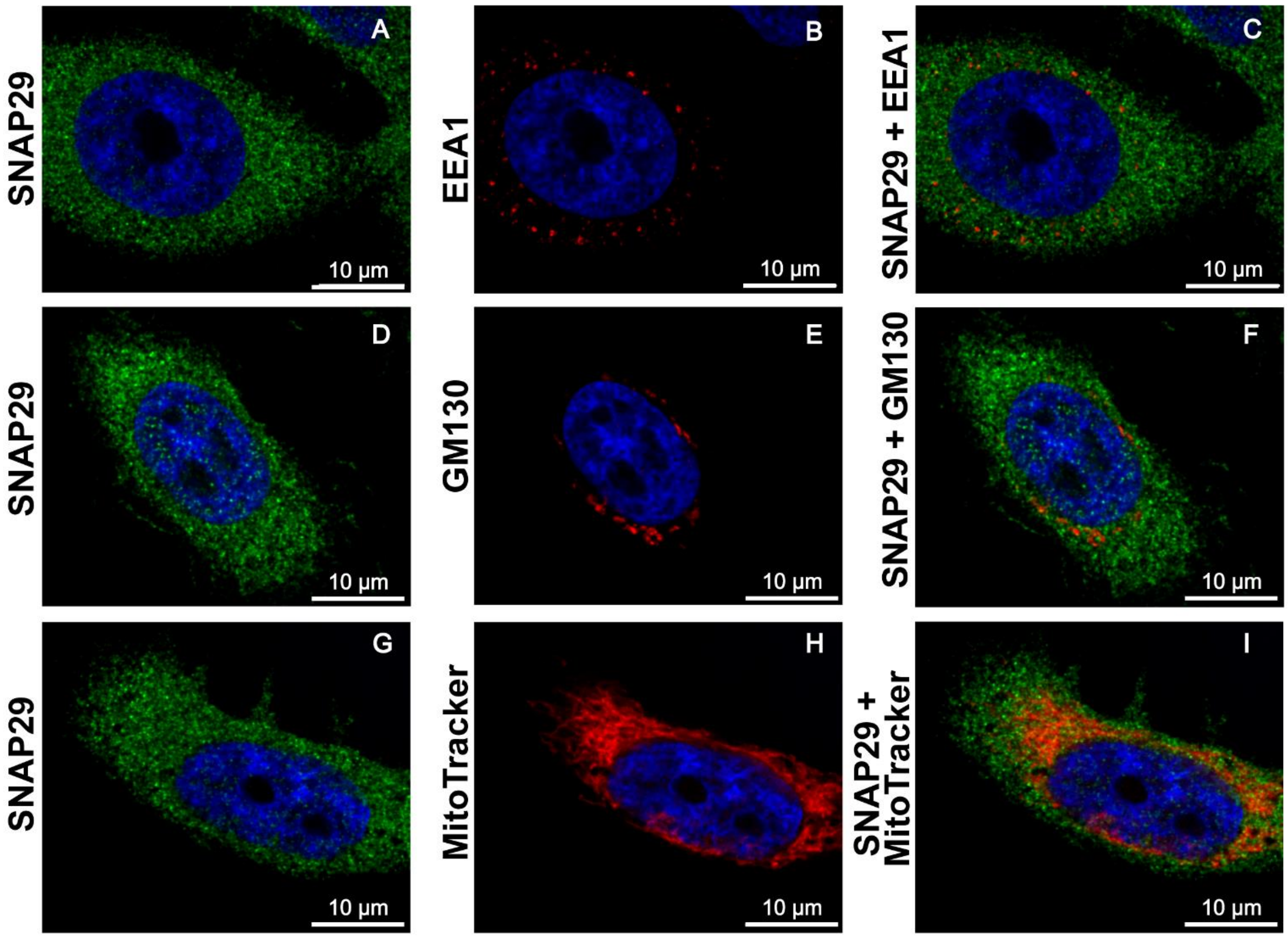

Supplement: Supplementary file 1 — Supplementary file1 (PDF 786 KB) [file 18_2023_4822_MOESM1_ESM.pdf]
